# Modelling seasonal habitat suitability for wide-ranging species: Invasive wild pigs in northern Australia

**DOI:** 10.1371/journal.pone.0177018

**Published:** 2017-05-04

**Authors:** Jens G. Froese, Carl S. Smith, Peter A. Durr, Clive A. McAlpine, Rieks D. van Klinken

**Affiliations:** 1 School of Agriculture and Food Sciences, The University of Queensland, St Lucia, Queensland, Australia; 2 School of Earth and Environmental Sciences, The University of Queensland, St Lucia, Queensland, Australia; 3 CSIRO Health and Biosecurity, Dutton Park, Queensland, Australia; 4 CSIRO Australian Animal Health Laboratory, East Geelong, Victoria, Australia; University of Sydney, AUSTRALIA

## Abstract

Invasive wildlife often causes serious damage to the economy and agriculture as well as environmental, human and animal health. Habitat models can fill knowledge gaps about species distributions and assist planning to mitigate impacts. Yet, model accuracy and utility may be compromised by small study areas and limited integration of species ecology or temporal variability. Here we modelled seasonal habitat suitability for wild pigs, a widespread and harmful invader, in northern Australia. We developed a resource-based, spatially-explicit and regional-scale approach using Bayesian networks and spatial pattern suitability analysis. We integrated important ecological factors such as variability in environmental conditions, breeding requirements and home range movements. The habitat model was parameterized during a structured, iterative expert elicitation process and applied to a wet season and a dry season scenario. Model performance and uncertainty was evaluated against independent distributional data sets. Validation results showed that an expert-averaged model accurately predicted empirical wild pig presences in northern Australia for both seasonal scenarios. Model uncertainty was largely associated with different expert assumptions about wild pigs’ resource-seeking home range movements. Habitat suitability varied considerably between seasons, retracting to resource-abundant rainforest, wetland and agricultural refuge areas during the dry season and expanding widely into surrounding grassland floodplains, savanna woodlands and coastal shrubs during the wet season. Overall, our model suggested that suitable wild pig habitat is less widely available in northern Australia than previously thought. Mapped results may be used to quantify impacts, assess risks, justify management investments and target control activities. Our methods are applicable to other wide-ranging species, especially in data-poor situations.

## Introduction

Where mammalian wildlife becomes invasive, it is often detrimental to the economy and agriculture as well as environmental, human and animal health [1,2]. To effectively mitigate impacts, spatially-explicit knowledge on invaders’ distribution and habitat use is needed [3,4]. This can be particularly challenging for wide-ranging species, as continuous empirical information is rarely available over broad geographic regions [5]. With rapid developments in spatial environmental data availability and new analytical methods, habitat models that infer species distributions from environmental predictor variables have proliferated to fill knowledge gaps [6,7]. Research is methodologically and terminologically diverse–depending on the research perspective, “habitat models” are also known as “species distribution models”, “ecological niche models”, “habitat suitability models”, “resource selection functions” and variations thereof [4,6–10]. However, important ecological considerations such as temporal variability or behavioural factors are often missed, especially in statistical, correlative models. This can affect model accuracy and utility for decision-making [11]. Here, we developed a resource-based, spatially-explicit approach to modelling seasonal habitat suitability for a widespread and harmful mobile invader, the wild pig (*Sus scrofa*), in northern Australia.

Wild pigs, originally native to Eurasia, are one of the most widespread terrestrial mammals [12]. Both wild and domesticated forms were introduced by early settlers to all continents and many oceanic islands [12]. In its introduced range, *S*. *scrofa* is also known as feral pig, feral swine, wild hog or razorback and has often been associated with severe negative impacts [12–14]. In Australia, invasive wild pigs are a major threat to unique ecosystems and agricultural industries [13,15]. They are most widespread in the tropical north, yet spatial knowledge is either empirical, detailed, and local scale [16–22], or expert-based, coarse, broad scale, and poorly validated [23–25]. Improved regional-scale knowledge of wild pig distribution could be used to delineate management units and limit re-invasion of conservation sites following local eradication [13,26]. It could also help assess the magnitude of environmental and economic impacts or the risk of establishment of infectious animal diseases, especially when abundance estimates are derived [27–30].

Statistical habitat models for wild pigs have been developed for northern Australia [31,32] as well as parts of Europe and North America with similar knowledge gaps [33–36]. However, some general limitations of statistical models are also apparent in these studies. First, correlative models calibrated from species presence or presence/absence records can only reliably predict species distribution within, and not outside, the environmental gradients used for model calibration [37]. Second, except for Morelle and Lejeune’s [36] study in Belgium, all models were calibrated from aggregate species records and did not consider temporal variability or ecological factors. In northern Australia such models may yield misleading results when reflecting previous research: Caley [16] and Hone [19] showed that wild pig distribution and abundance varies considerably between the wet and dry season; Caley [17] and Mitchell *et al*. [21] found that habitat use and home range movements differ distinctly between breeding herds (consisting of related sows and their young) and solitary boars; and Choquenot and Ruscoe’s [38] work suggested that wild pig persistence depends on complementary access to key resources within the boundaries of such home ranges.

Here we adapted a resource-based modelling framework using Bayesian networks that allowed us to address these issues. This general approach has previously been applied to habitat models [39–42] and offers several advantages over correlative methods: a robust statistical framework for modelling interactions between habitat variables based on species ecology rather than distributional data; flexible data requirements with the ability to integrate unpublished expert knowledge; and explicit treatment of the uncertainty in parameter estimates [43–45]. Our objectives were: (1) to model seasonal habitat suitability for wild pig breeding and persistence in northern Australia at the regional scale whilst integrating behavioural factors as well as temporal variability; and (2) to rigorously evaluate accuracy and uncertainty in our expert-elicited models by validating spatial predictions of habitat suitability against truly independent distributional data sets.

## Materials and methods

### Study region

**Fig 1:**
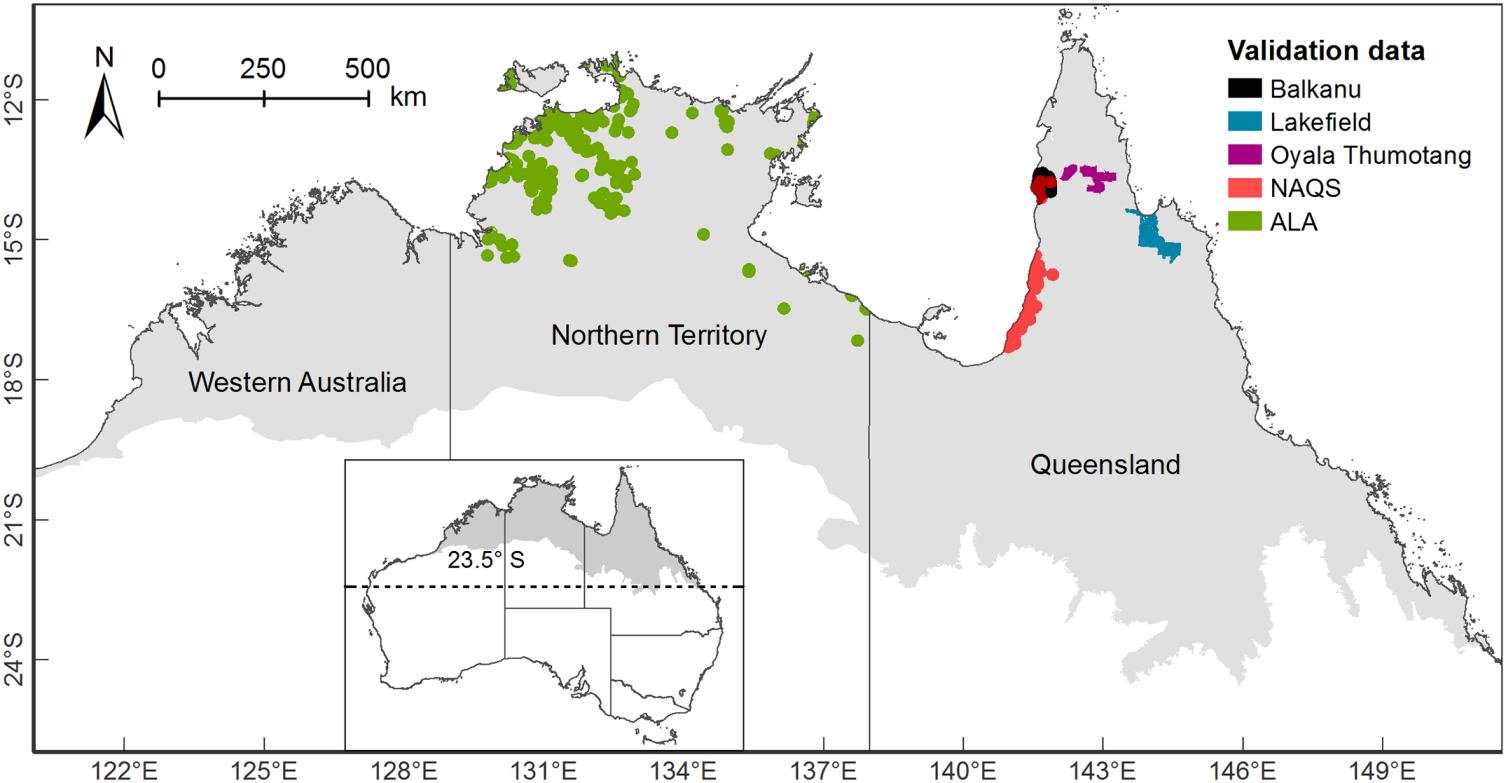
Study region in northern Australia. The study region is shown in grey. Locations of the independent distributional data sets used for model validation are shown in colour.

Our study region covered 1.76 million km^2^ north of the Tropic of Capricorn spanning three Australian states (Fig 1). The climate is tropical with seasonal rainfall, alternating between a wet and a dry season. Rainfall and primary productivity broadly decline on a north-south, and to a lesser extent, on an east-west, gradient [46,47]. Monsoonal savanna woodlands and semi-arid grasslands are interspersed with riverine channels, seasonally inundated floodplains, coastal wetlands and rainforest fragments [48]. Intensive human uses are concentrated in fertile coastal lowlands. The semi-arid inland is sparsely populated.

At a coarse scale, all of the study region appears climatically suitable for wild pigs and has mostly been invaded [24]. Arid regions with insufficient rainfall were not included in our study. Wild pigs are reported to be widespread in the east and localised in the north and west [24]. Highest local densities have been recorded in resource-abundant wetlands and floodplains, yet these populations fluctuate considerably with climatic conditions [14,16,19]. A wide range of management activities are conducted throughout northern Australia to mitigate wild pig impacts, including lethal and non-lethal methods. Yet, effective management is hampered by the region’s remoteness and there is little evidence of sustained population reduction [13].

### Habitat suitability model

**Fig 2:**
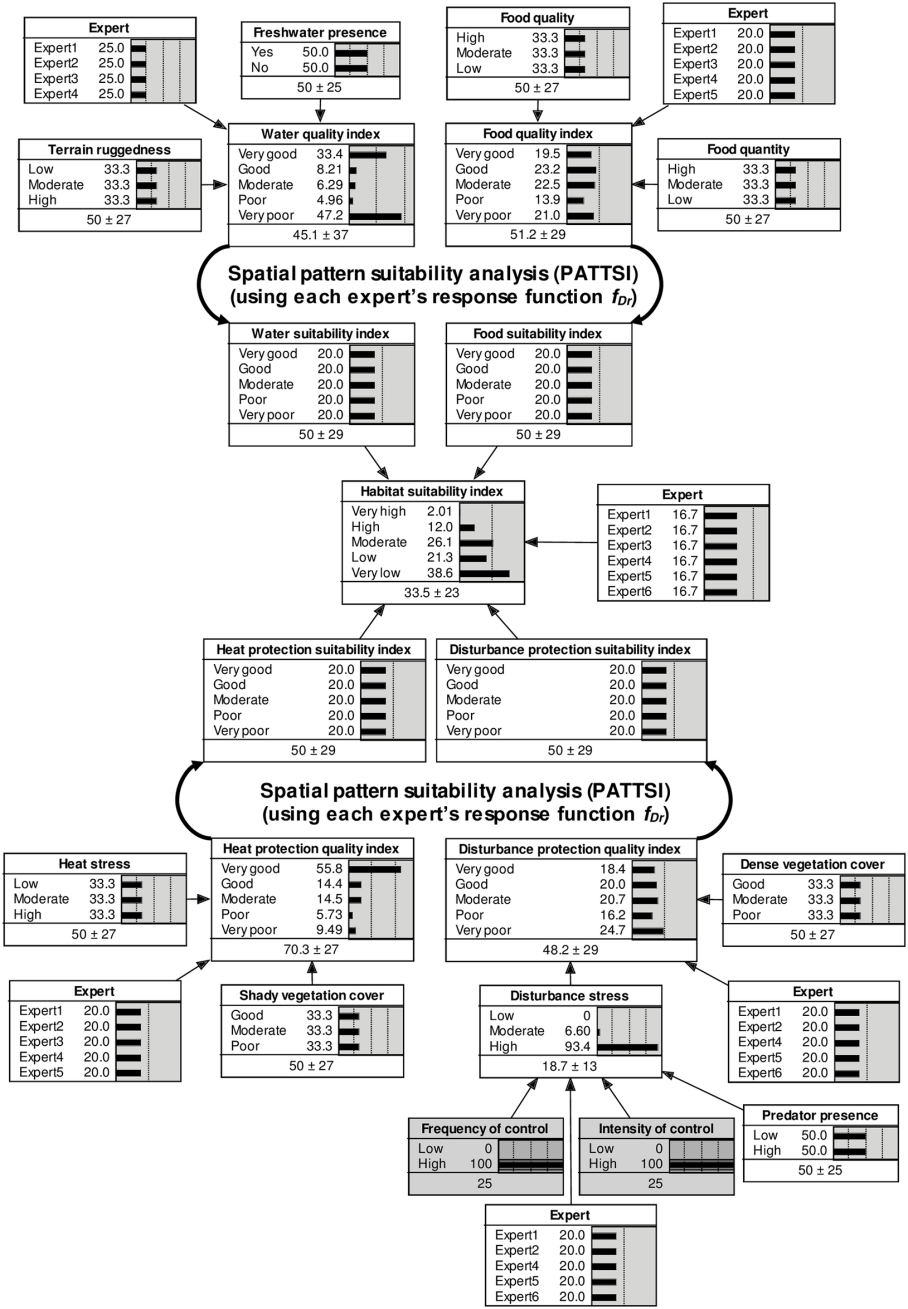
Wild pig habitat suitability model. Resource quality indices for each habitat variable were modelled in Bayesian networks. Spatial pattern suitability analysis was used to compute resource suitability indices as a weighted function of distance to resource patches (*f*_*Dr*_). Habitat suitability was modelled in another Bayesian network. An average habitat suitability index was computed and mapped from six individual expert models. Bar graphs show expert-elicited conditional probabilities and values below graphs show modelled index values ± standard deviation. Probabilities and indices change once evidence about the states of each explanatory variable at a given study area pixel is inserted (i.e. prior probabilities are no longer uniformly distributed).

Our modelling approach consisted of three main steps (Fig 2). First, we modelled ‘resource quality indices’ for a suite of habitat variables, referenced specifically to the resource requirements of wild pig breeding herds, in separate Bayesian networks. Second, we used ‘spatial pattern suitability analysis’ to capture wild pigs’ ability to access complementary resources at different locations within their home range. Finally, we modelled a ‘habitat suitability index’ by combining all ‘resource suitability indices’ in another Bayesian network. The model was calibrated using a structured, iterative elicitation process [49] with a panel of experts. Experts were practitioners with field knowledge of wild pigs from various localities and professional backgrounds [50]. We combined techniques for eliciting system understanding through group consensus and for eliciting quantitative estimates from individuals with opportunities for Delphi-style revision [42,51–53]. Expert elicitation was approved by the *CSIRO Human Ethics Committee* (Project 075/13) and written consent obtained from all participants.

#### Bayesian network models

We adapted the Bayesian network modelling framework proposed by Marcot *et al*. [39], refined by Smith *et al*. [40] and explained in detail by van Klinken *et al*. [42]. Here, habitat suitability was conditional on a set of habitat variables representing resource requirements. Each habitat variable was itself influenced by several measurable key explanatory variables, and each explanatory variable was linked to one or more remotely sensed or mapped spatial data proxies. Our model was implemented in the *Norsys Netica v*.*5*.*12* software.

#### Expert elicitation

During an initial expert workshop, a preliminary model was developed [49]. A panel of experts (n = 18) constructed a conceptual model, defined each model variable (habitat suitability, habitat variables and explanatory variables including spatial data proxies) and assigned it mutually exclusive states. We quantified causal relationships in the network by eliciting conditional probability tables (CPTs) behind each response variable (child node). We used the *CPT calculator* software [54], which reduces the number of elicited response probabilities to key scenarios, i.e. combination of states in explanatory variables (parent nodes), and interpolates all other combinations. Each step was performed in break-out groups or individually, followed by panel discussion and consensus formation (except for the *CPT calculator*) [42,53].

Following preliminary application, sensitivity analysis and validation of the preliminary model, we conducted semi-structured interviews with a self-selected subsample from our panel of experts (n = 6). Model structure, spatial data proxies and evaluation results were reviewed against each expert’s knowledge and simplified CPTs were parameterized. We asked experts to revise prior CPTs from the preliminary model rather than parameterising new ones [51,52]. As interviews were less time-constrained than the workshop, experts could utilize either or both of two elicitation methods that were more flexible and robust to error than the *CPT calculator* [54]. Method A was implemented in the *AgenaRisk v*.*6*.*1* software and made the simplifying assumption that any response follows a truncated normal distribution (*TNormal*) centred on the weighted mean of its explanatory variables [55]. In order to use this method, we converted all model variables into “ranked nodes”, whose states were assigned with equal intervals on a numerical scale from 0 to 100 [55]. Experts only defined: (a) the weight of each explanatory variable, (b) overall uncertainty in making this judgement (determining the variance of *TNormal*), and (c) whether the weighted mean function should be replaced by either a weighted minimum (to describe limiting factor relationships), or maximum (to describe substitution relationships) function [55]. Method B restricted elicitation to key scenarios as in [54]. However, instead of directly assigning probabilities to each state of the response variable, we used interval judgements [56,57], asking experts for their best estimate and the outer bounds of a 95% confidence interval. To maintain consistency with method A, we allowed only *TNormal* distributions centred on the best estimate. Post-elicitation, we discretised interval judgements into probabilities for each response state using a binning algorithm.

#### Habitat suitability and resource quality indices

The final Bayesian network model [49] is shown in Fig 2. Expert-elicited CPTs, definitions for all model variables and their states and spatial data proxies that determined the state of each explanatory variable at each pixel in the study region are provided as supporting information (S1 and S2 Tables). Experts identified four key resource requirements for sustained wild pig breeding: food, water, protection from heat and protection from disturbance (Fig 2). Each of these habitat variables had five states with corresponding equal numerical intervals (0–20 for the poorest state, …, 80–100 for the best state). For each habitat variable, we computed expert-averaged ‘resource quality indices’ (*x*_*r*_) as model expected values from an equal-weighted average CPT [52,58] (Fig 2) by summing the mid-point value of each state interval weighted by its probability. Accordingly, *x*_*r*_ could range between 10 (mid-point of the poorest state) and 90 (mid-point of the best state) and varied spatially according to the combination of states of explanatory variables at a given pixel. Following spatial pattern suitability analysis of pixel-scale *x*_*Water*_, *x*_*Food*_, *x*_*Heat*_ and *x*_*Disturbance*_ (see below), we computed a ‘habitat suitability index’ (*HSI*) from the derived landscape-scale habitat variables [59,60]. The method was analogous to the resource quality indices. However, we used each individual expert’s CPT to compute model expected *HSI*. This allowed us to evaluate average results as well as model uncertainty from diverging expert knowledge.

#### Spatial pattern suitability analysis

In order to capture wild pigs’ ability to access their four resource requirements at different locations within heterogeneous home ranges, we converted pixel-scale resource quality indices (*x*_*r*_) into landscape-scale ‘resource suitability indices’ (*SI*_*r*_). Apart from various empirical estimates of female home range sizes (1–20 km^2^ in [14,61]) and a previous finding that pasture and riverine woodlands must co-occur within 5 km [38], we had limited *a priori* knowledge about wild pigs’ resource-seeking home range movements in northern Australia.

**Fig 3:**
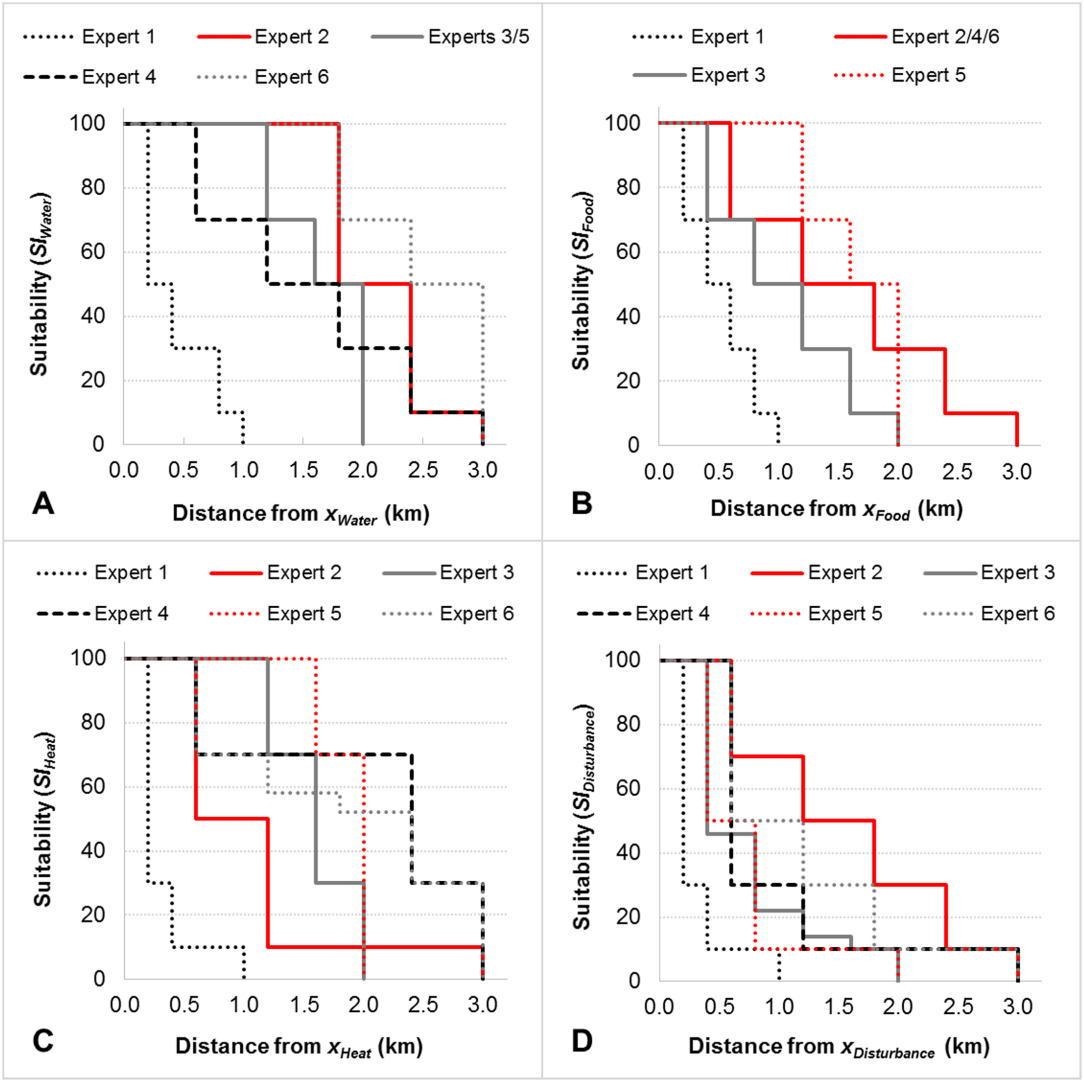
Expert-elicited resource suitability in response to distance. Resource suitability indices (*SI*_*r*_) were computed from elicited response-to-pattern curves (*f*_*Dr*_) for each of the four habitat variables in the model: water (A), food (B), heat protection (C) and disturbance protection (D). Curves cross the x-axis at different points because experts defined different home range boundaries.

We therefore elicited distance-dependent response-to-pattern curves (*f*_*Dr*_) for each habitat variable from individual experts (n = 6) during interviews (Fig 3 and S1 Appendix). This involved first specifying a wild pig ‘mobility threshold’ (or home range boundary) beyond which resources are inaccessible to breeding herds. Experts defined these at 1 km (n = 1), 2 km (n = 2) or 3 km (n = 3), corresponding to circular home ranges of approximately 3, 12 and 28 km^2^. Second, experts described the functional value of a given resource for wild pig breeding in response to distance (Fig 3). Third, we applied spatial moving window analysis to compute *SI*_*r*_ at a focal pixel as the highest distance-weighted *x*_*r*_ of all resources contained within an analysis window. This window was shifted and centred on each pixel within the study region. We used circular moving windows [59] with radii defined by elicited mobility thresholds and distance weights defined by response curves *f*_*Dr*_. We termed this combined methodology ‘spatial pattern suitability analysis’ (S1 Appendix).

#### Seasonal scenarios

We applied each individual expert’s final model [49] to two seasonal scenarios by linking model explanatory variables to seasonally-specific spatial data proxies (Table 1). We were most interested in the late wet season (March to April), when resources required by wild pigs are abundant and widely distributed, and the late dry season (October to November), when resources are scarce and scattered across the region. Suitable remotely sensed or mapped spatial proxies were: (a) discussed with experts and sourced from various agencies (S2.2 Table); (b) rasterized and resampled to a common extent and a fine resolution (1 ha) for capturing spatial heterogeneity relevant to wild pigs [42]; and (c) averaged for the two months corresponding to each seasonal scenario over five years (2010–2014) to reflect average conditions. Finally, spatial data attributes were reclassified to match the states of explanatory variables (Table 1 and S2.2–S2.6 Tables). Some model variables were linked to static spatial proxies without seasonal variability (e.g. terrain ruggedness). For three variables determining *Disturbance stress* (Fig 2), no spatial proxies were available for the study region. We applied a global (spatially uniform) scenario [42], with “high” *Intensity of control* and *Frequency of control* and no selected state for *Predator presence* (Fig 2). While this assumption likely overestimated disturbance in our models, it approximated conditions under which most of the local validation data were collected.

**Table 1:** Model explanatory variables and spatial data proxies.

Explanatory variable	Seasonal	Spatial data proxies	Classification or thresholding methods
Freshwater presence	Yes	Queensland wetland data Geofabric Surface Cartography Present Major Vegetation Subgroups	Presence and seasonality from data attributes Rainforests classed as perennial freshwater (because water bodies inadequately mapped)
Terrain ruggedness	No	3 sec SRTM derived Digital Elevation Model	Thresholds (from terrain ruggedness index) estimated from map
Food quality	Yes	Present Major Vegetation Subgroups Catchment scale Land Use of Australia Monthly relative soil moisture upper layer (WRel1)	Protein accessibility thresholds (from average WRel1) estimated from map Food quality class under good/poor protein accessibility elicited from experts
Food quantity	Yes	Monthly fractional cover of Photosynthetic Vegetation (f_PV_)	Vegetative productivity thresholds (from average f_PV_) assigned from literature
Heat stress	Yes	Monthly mean maximum temperature	Thresholds (average T_max_) elicited from experts
Shady vegetation cover	No	Persistent Green Vegetation Fraction 2000–2010	Foliage cover thresholds assigned from literature
Dense vegetation cover	No	Present Major Vegetation Groups Catchment scale Land Use of Australia	Density class elicited from experts

Further detail about spatial data proxies including references and methods for reclassifying data attributes into state-specific categories is provided in S2.2–S2.6 Tables.

### Model evaluation and validation

#### Sensitivity analysis

Behaviour of each individual expert model as well as an expert-averaged consensus model was evaluated using the “Sensitivity to findings” algorithm in the *Norsys Netica 5*.*12* software. We focussed on the variance reduction metric recommended for numerical variables [62]. It assessed the relative influence of our four habitat variables on predicted habitat suitability by calculating how much the variance of *HSI* was reduced by entering a particular finding (i.e. *SI*_*r*_ value) for one of the habitat variables. Greater variance reduction means that *HSI* was more sensitive to a change in the state of the habitat variable [62,63].

#### Predictive performance

Predictive model performance was validated against four data sets of wild pig presence per seasonal scenario (Table 2). Most data were independently collected by external agencies in conjunction with aerial management activities. As aerial survey data was only available for the eastern state of Queensland (Fig 1), we also obtained presence records (search term “*Sus scrofa*”) from the national *Atlas of Living Australia*. This database contained only one dated record in the Western Australian portion of our study area. Hence, we confined the downloaded ‘ALA’ dataset to records from the Northern Territory, which appeared adequately sampled (748 unfiltered records). Where possible, we matched data to model assumptions, using only presence records that corresponded to breeding herds (identified as female or with a group count greater than two) and were dated in the late wet and late dry season respectively. To reduce unwanted noise from spatial error in validation data or spatial proxies used for modelling [64,65], we upscaled both predicted *HSI* and wild pig presence records to a 1 km resolution. We subsequently thinned data to ensure independence among data points, allowing only one data point collected on the same day within a given 1 km pixel.

**Table 2:** Validation data sets with ancillary information.

Name	Source	No. of records	Date of collection	Method/ purpose of collection	Background size (km^2^)	Typical habitat types
Balkanu	Balkanu Cape York Development Corporation	dry: 181 wet: 67	Sep-Nov 2013–14 May 2015	Systematic aerial survey/ management (shooting)	dry: 3,954 wet: 3,089	Eucalyptus woodlands & coastal wetlands
Lakefield	Queensland Parks & Wildlife Service	dry: 350 wet: 124	Oct-Dec 2009–13 Feb-May 2010–13	Systematic aerial management (shooting)	5,788	Eucalyptus/ Melaleuca woodlands, coastal wetlands & grasslands
Oyala Thumotang	Queensland Parks & Wildlife Service	wet: 263	Apr-May 2010–13	Systematic aerial management (shooting)	3,819	Eucalyptus woodlands, riparian Melaleuca forests & rainforests
ALA	Atlas of Living Australia	dry: 111 wet: 144	Sep-Dec 98–2012 Feb-May 91–2012	Surveys/ sightings (purpose unknown)	dry: 36,024 wet: 41,511	Eucalyptus woodlands, floodplains, Melaleuca forests & mangroves
NAQS	Northern Australia Quarantine Strategy	dry: 103	Sep-Nov 2007–10	Opportunistic aerial survey / disease sampling (shooting)	11,630	Eucalyptus woodlands, floodplain grasslands & chenopod scrublands

For each data set, we also defined a validation background, which served to contrast presences with areas from which feral pips could be considered ‘absent’ (Fig 1). In doing so, we aimed to strike a compromise between (a) evaluating performance across sufficiently large areas to justify inferences about the models’ discriminatory power and (b) restricting evaluation to similar environmental gradients as contained in the presence data so that validation metrics are not artificially inflated [66]. Where possible, we defined backgrounds from existing management units in which surveys were conducted (National Park boundaries for ‘Lakefield’ and ‘Oyala Thumotang’). Otherwise, we arbitrarily applied a 15 km buffer to data points (for ‘Balkanu’, ‘NAQS’ and ‘ALA’), which corresponded to five times the highest expert-elicited mobility threshold (home range boundary) in this study.

We used the *Continuous Boyce Index (CBI)* to evaluate model performance (S2 Appendix). This method was developed specifically for evaluation against presence-only observations [64,67]. A *predicted-to-expected* (*P/E*) *ratio* was computed as the (predicted) proportion of presence records coinciding with a specified range of *HSI* values (bin) divided by the (expected) proportion of the validation background covered by that bin. The *CBI* measures the Spearman rank correlation coefficient of *P/E* against *HSI* and varies from 1 (correct model, *P/E* steadily increases as *HSI* increases) to –1 (false model, *P/E* steadily decreases with increasing *HSI*). Values close to zero indicate a random prediction [67]. We computed the *P/E ratio* and *CBI* for each expert model / validation data combination. We also computed the proportion of each validation background expected to be highly or very highly suitable (*HSI* ≥ 60). This resulted in 28 sets of validation metrics (*P/E ratio* + *CBI* + *HSI* ≥ 60) per seasonal scenario (6 expert models x 4 validation data + 1 expert-averaged model x 4 validation data, S2 Appendix). This allowed us to evaluate uncertainty from diverging expert knowledge and test the expectation that an expert-averaged model performs best [58].

## Results

### Sensitivity analysis

**Fig 4:**
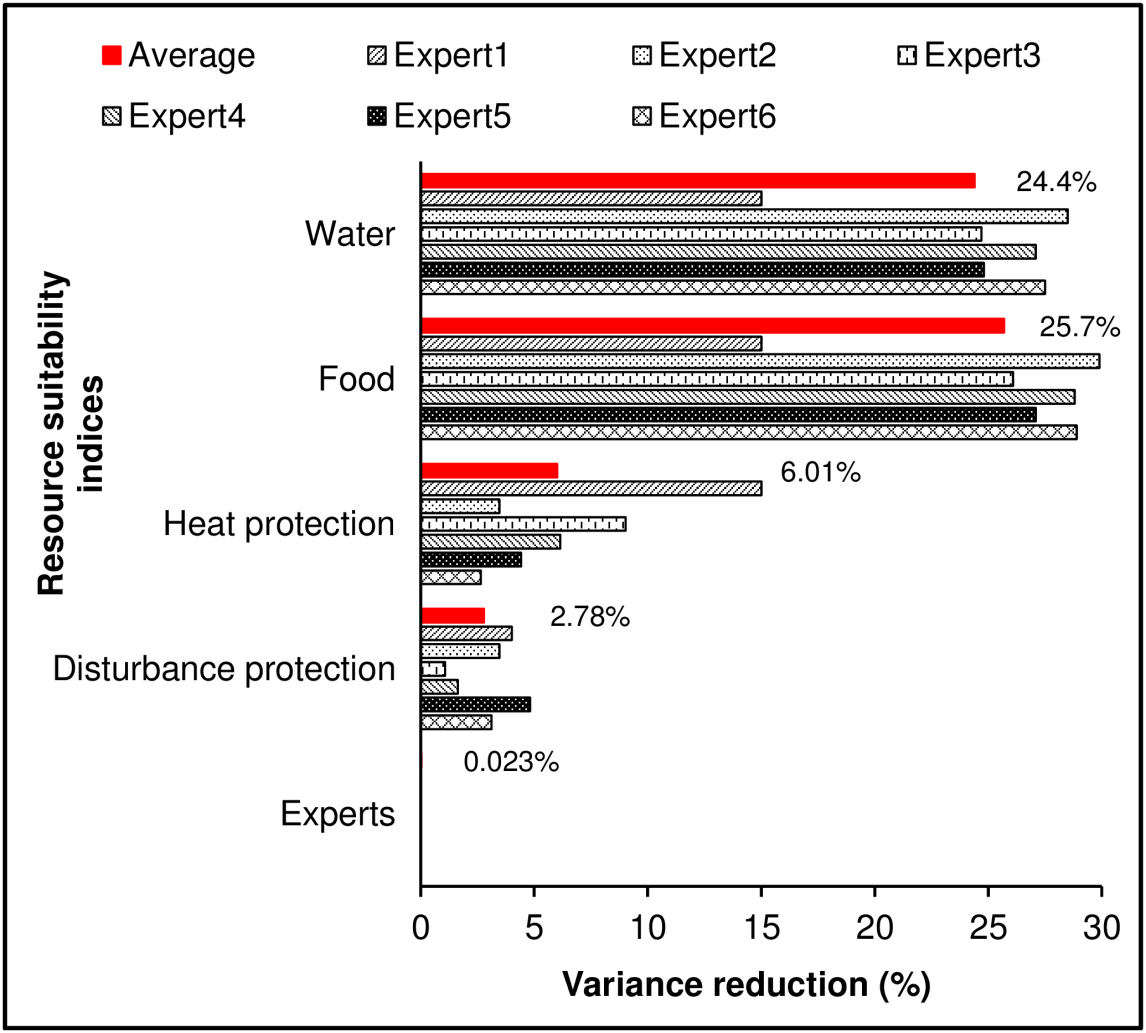
Sensitivity of habitat suitability to four habitat variables and expert opinion. Sensitivity to findings was calculated as % variance reduction for each individual expert model and an averaged model (red bar and percentages).

Sensitivity analysis revealed that habitat suitability in the Bayesian network model was most strongly influenced by water and food resources (24.4% and 25.7% variance reduction respectively) and to a lesser degree by protection from heat (6.01%) and protection from disturbance (2.78%) (Fig 4). Habitat suitability was least sensitive to expert opinion (0.023% variance reduction). Hence, experts were mostly in agreement about the relative importance of the four habitat variables and quantified model relationships similarly. However, one expert weighted the heat protection requirement as highly as water and food resources (Expert 1: 15% variance reduction, Fig 4).

### Predictive performance

**Fig 5:**
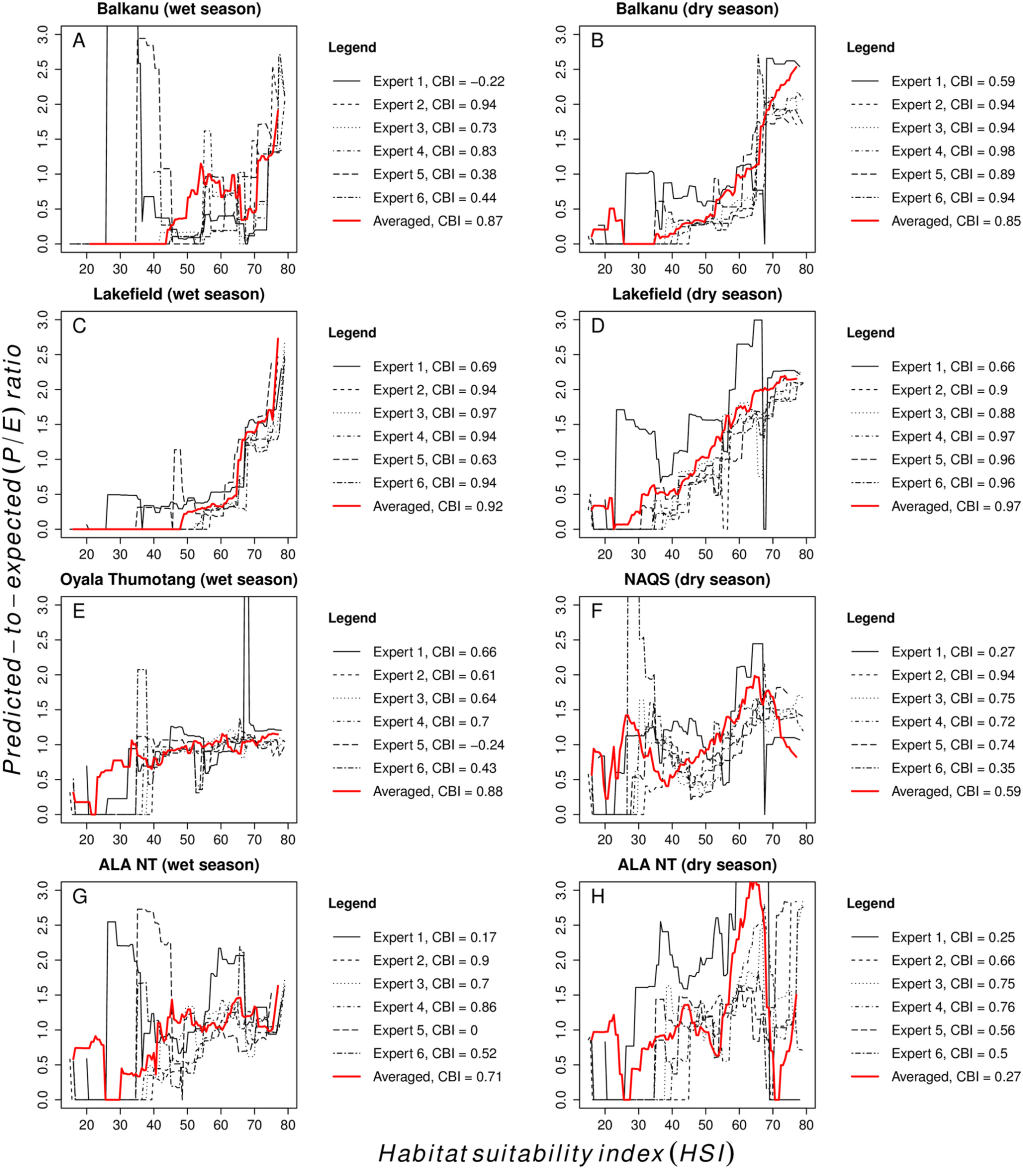
Validation plots for individual expert and averaged seasonal habitat suitability models. Validation was performed against four validation data sets per seasonal scenario (Table 2). The *predicted-to-expected (P/E) ratio* (y axis) measures the proportion of wild pig presences relative to the proportion of background pixels on a continuous scale of predicted *HSI* (x axis).

In general, expert-elicited habitat suitability models performed well against the validation data (Table 3 and Fig 5). For some seasonal scenario / validation data combinations, all expert models performed well (e.g. wet or dry season model / Lakefield data). For others, there were considerable differences (e.g. wet or dry season model / ALA data) (Table 3). As the *HSI* Bayesian network model was not sensitive to expert opinion, model uncertainty stemmed largely from disagreement about wild pigs’ resource-seeking home range movements, i.e. expert-elicited response-to-pattern curves (Fig 3). Expert models that assumed high mobility thresholds (3 km for experts 2, 4 and 6, Fig 3) predicted the highest proportions of suitable habitat in all validation backgrounds. Average *HSI ≥ 60* for these three expert models ranged between 71–78% in the wet season and 36–42% in the dry season. Model accuracy was also generally highest for these experts, with average *CBI* ranging between 0.58–0.85 in the wet season and 0.69–0.86 in the dry season. Both metrics were lowest for the expert model that assumed the least mobility (1 km for expert 1, Fig 3). Here, average *HSI ≥ 60* was 47% (wet) and 15% (dry) and average *CBI* was 0.33 (wet) and 0.44 (dry).

**Table 3:** Validation metrics for individual expert and averaged seasonal habitat suitability models.

Habitat suitability model	Validation metrics per model scenario / validation data combination															
	Wet season scenario								Dry season scenario							
	Balkanu		Lakefield		Oyala Thum		ALA		Balkanu		Lakefield		NAQS		ALA	
	*CBI*	*HSI60*	*CBI*	*HSI60*	*CBI*	*HSI60*	*CBI*	*HSI60*	*CBI*	*HSI60*	*CBI*	*HSI60*	*CBI*	*HSI60*	*CBI*	*HSI60*
Expert 1	-0.22	59%	0.69	60%	0.66	41%	0.17	26%	0.59	28%	0.66	15%	0.27	12%	0.25	3%
Expert 2	0.94	83%	0.94	91%	0.61	77%	0.9	58%	0.94	64%	0.9	38%	0.94	44%	0.66	20%
Expert 3	0.73	80%	0.97	85%	0.64	68%	0.7	54%	0.94	58%	0.88	30%	0.75	44%	0.75	15%
Expert 4	0.83	82%	0.94	83%	0.7	68%	0.86	49%	0.98	56%	0.97	33%	0.72	42%	0.76	12%
Expert 5	0.38	74%	0.63	56%	-0.24	69%	0	42%	0.89	50%	0.96	34%	0.74	30%	0.56	9%
Expert 6	0.44	83%	0.94	91%	0.43	77%	0.52	60%	0.94	63%	0.96	38%	0.35	44%	0.5	22%
Averaged	0.87	71%	0.92	70%	0.88	59%	0.71	39%	0.85	44%	0.97	26%	0.59	25%	0.27	7%

Validation was performed against four data sets per seasonal scenario (Table 2). We show the *Continuous Boyce Index* (*CBI*) and proportion of validation background expected to be highly or very highly suitable habitat (*HSI ≥ 60*, here shortened to *HSI60*). A *CBI* = 1 would indicate a perfectly accurate, a *CBI* ~ 0 a random, and a *CBI* < 0 a false model.

The expert-averaged model performed similar to, or better than, the best individual expert models for most validation data, except for the dry season model / NAQS data and dry season model / ALA data combinations. Its average *CBI* across all validation data sets was 0.85 in the wet season and 0.67 in the dry season. The predicted proportion of suitable habitat was modest compared to individual expert models (except for expert 1), with an average *HSI ≥ 60* of 60% (wet) and 26% (dry). While averaging did not increase model accuracy as expected, it produced consistently accurate results for all validation data (Table 3, Fig 5 and S1 Fig).

### Seasonal habitat suitability

**Fig 6:**
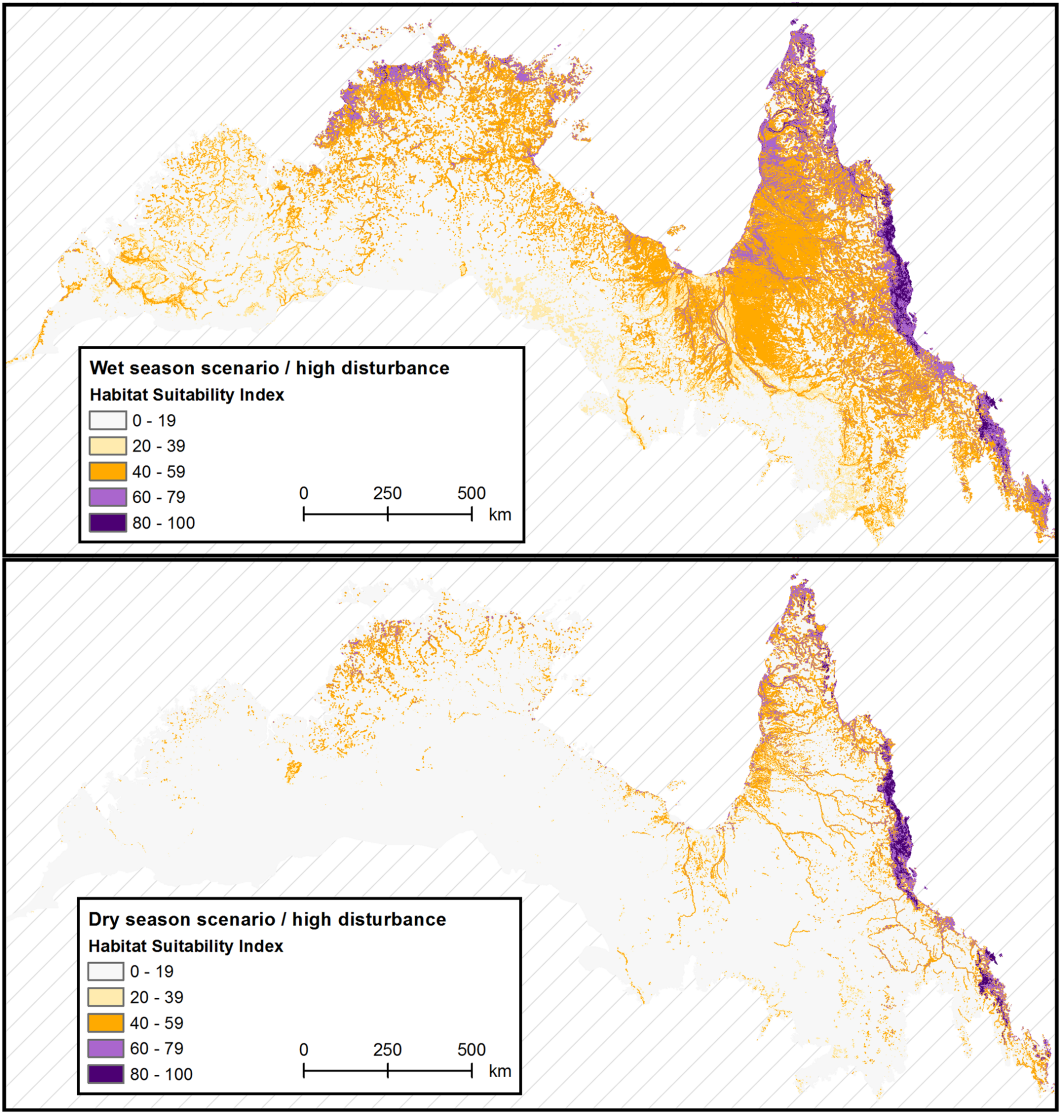
Seasonal habitat suitability for wild pig breeding in northern Australia. Habitat suitability indices were averaged across all expert models and mapped for a wet (March/April–A) and dry (October/November–B) season scenario.

We present and discuss seasonal results only for the expert-averaged consensus model, which produced consistently accurate results across the study region. Predicted habitat suitability varied considerably between seasonal scenarios (Table 4 and Fig 6). Overall the model predicted suitable habitat (*HSI* ≥ 40) in 36.2% of the study region (~640,000 km^2^) during the wet season and 9.5% (~170,000 km^2^) during the dry season. Of this, about one quarter was highly or very highly suitable habitat (*HSI* ≥ 60, 8.4% during the wet season and 2.8% during the dry season, Table 4).

**Table 4:** Amount of wild pig habitat in each habitat suitability index class per seasonal scenario.

Seasonal scenario	Total area covered by each habitat suitability index class (km^2^ and %)				
	Very high	High	Moderate	Low	Very low
Wet season	16,726 (1%)	130,112 (7.4%)	489,371 (27.9%)	239,948 (13.7%)	880,902 (50.1%)
Dry season	11,147 (0.6%)	39,512 (2.2%)	116,273 (6.6%)	71,532 (4.1%)	1,518,597 (86.4%)

**Fig 7:**
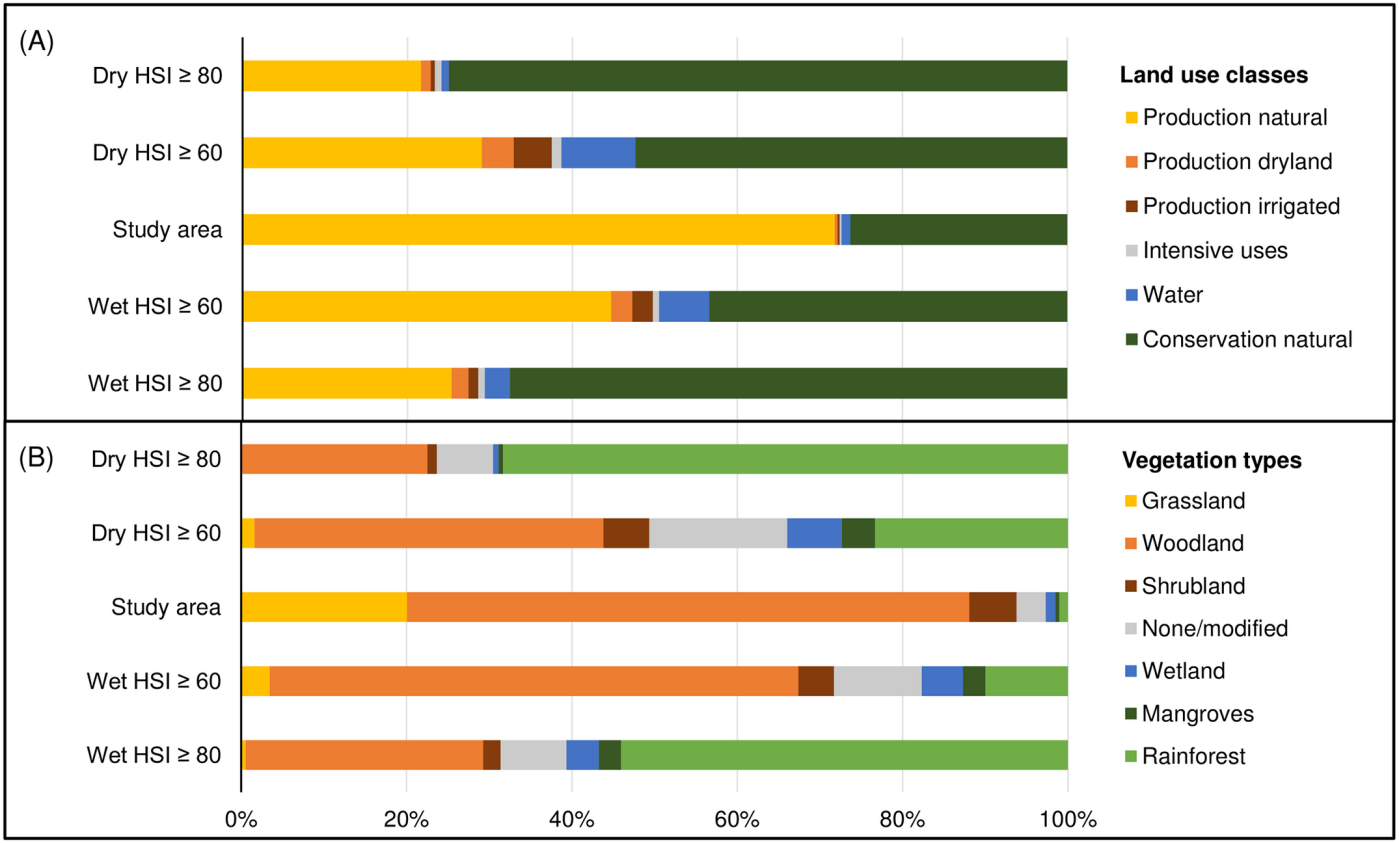
Share of modelled suitable habitat found in different vegetation types and land use classes for each seasonal scenario. For each vegetation (panel A) or land use (panel B) category, we show its percent share of highly (*HSI ≥ 60*) and very highly (*HSI ≥ 80*) suitable habitat during the dry (top bars) or wet (bottom bars) season, compared to its percent share of the total study region (central bars). Spatial analyses in panel (A) were based on *Present Major Vegetation Groups* (MVG V.4.1) and analyses in panel (B) on *Australian Land Use and Management* classes (ALUM V.7).

Habitat suitability also varied spatially between administrative units, vegetation types and land use classes (Figs 6 and 7 and S2 Fig).

When analysed by states (shown in Fig 1), highly and very highly suitable wild pig habitat was located mostly in Queensland, especially during the dry season. It was largely absent from Western Australia in either scenario. The Northern Territory’s share increased more than three-fold during the wet season. Moderately suitable habitat (40 ≤ *HSI* < 60) was somewhat more evenly distributed across the study region, especially during the wetter months (covering about 40% of the study region in Queensland, 20% in the Northern Territory and 10% in Western Australia, S2.1 Fig). Within each state, suitable habitat was concentrated in coastal environments during the dry season (except for some large inland riverine and wetland systems), and expanded widely during the wet season (Fig 6).

By broad vegetation types [68] (Fig 7), highly suitable habitat (*HSI* ≥ 60) was disproportionately found in rainforests, wetlands, mangroves and modified (agricultural) vegetation, especially during the dry season (23%, 7%, 4% and 17% share of total suitable habitat respectively). Yet, these vegetation types covered only 6.2% of the study region combined. A large share of suitable habitat– 42% (dry) and 64% (wet)–was also contained in the region’s dominant vegetation type, savanna woodlands, which was broadly proportional to its overall cover (68% of the study region). Very highly suitable habitat was even more concentrated in rainforests (68% (dry) and 54% (wet) share of *HSI* ≥ 80) and less frequently found in savanna woodlands (22% (dry) and 29% (wet) share of *HSI* ≥ 80, Fig 7). During the dry season, the vast majority of grasslands (98%), shrublands (93%) and woodlands (91%) were modelled as unsuitable for wild pig breeding (*HSI* ≤ 40, S2.2 Fig). During the wet season, habitat concentration was weaker and suitable habitat was somewhat more evenly distributed among vegetation types (Fig 7).

Habitat suitability was also unevenly distributed between land use classes [69] (Fig 7). During the dry season, high value land used for water resources, irrigated or dryland production together contained 17% of suitable habitat on less than 2% of the study region. Interestingly, the 26% of the study region set apart for nature conservation also contained a disproportionate amount of suitable habitat (52% (dry) and 43% (wet) share of *HSI* ≥ 60). The dominant land use type “grazing natural vegetation” contained a greater share of suitable habitat during the wet season (42%) than the dry season (24%) on 71% of the study region. As with vegetation types, habitat concentration was further increased for very highly suitable habitat (*HSI* ≥ 80) and weaker in the wet than in the dry season (Fig 7).

## Discussion

Effective management of invasive wildlife requires spatially-explicit knowledge of their distribution and habitat use, especially for wide-ranging species [4]. Yet, continuous empirical information is rarely available over large areas [5]. Habitat models can fill this knowledge gap, but their utility may be compromised by small study areas and limited integration of species ecology or temporal variability [11]. Here, we modelled, for the first time, seasonally-specific habitat suitability for wild pigs, a widespread and harmful invader, in northern Australia. Rigorous evaluation and validation showed that our resource-based, expert-elicited model, which integrated important ecological factors such as home range movements and breeding requirements, accurately predicted wild pig presence across the study region. Results suggest that suitable wild pig habitat is more constrained in northern Australia than previously thought, especially during the dry season. Mapped results may be used by land managers to quantify impacts, assess risks, justify investments and target control activities. Our transparent methodology could be applied to other wide-ranging species, especially in data-poor situations.

### Seasonal habitat suitability

Our habitat model confirmed previous site-scale findings that the distribution and habitat use of wild pigs in northern Australia is highly seasonal [14,16,19,70] for the entire region. Modelled habitat suitability for wild pig breeding and persistence was mainly driven by seasonal availability of food and water resources, both of which ultimately vary with northern Australia’s annual rainfall cycle. Our model indicated a four-fold increase in suitable habitat during the wet season. Inter-annual climatic variability, which has been shown to greatly affect wild pig populations in drier parts of Australia [70–72], was not investigated in this study. Our scenario approach could be usefully extended to model cyclical, as well as seasonal, variability in wild pig distribution.

Seasonal fluctuations in habitat suitability were expressed at different spatial scales. At the regional scale, habitat suitability varied along an east-west gradient. Suitable wild pig habitat was concentrated in the eastern study region throughout the year. During the dry season, suitable habitat contracted more in the west than in the east (eleven-fold in Western Australia, five-fold in the Northern Territory and three-fold in Queensland). These patterns correspond well with prevailing rainfall gradients and harsher dry season conditions in the west [46,47]. At the landscape scale, contiguous patches of suitable habitat were located predominantly along the coastline during the dry season and expanded widely across the study region during the wet season. If all suitable wet season habitat was to be used by wild pigs, this points to long distance seasonal migration. However, such migratory behaviour in Australian wild pigs has been rejected by previous research [17,21,61]. Rather, empirical findings suggest that wild pigs may expand and contract their home range in response to changing conditions [21,73], or shift it entirely if adverse conditions persist [14,17]. Hence, not all suitable wet season habitat may be within reach of wild pig breeding herds dispersing from dry season refuges [17]. At the local scale, dry season habitat was concentrated in productive rainforest, wetland and mangrove ecosystems as well as high value agricultural lands, where resources remain abundant. While these dry season refuges continued to provide suitable habitat during the wet season, habitat was more evenly distributed among vegetation types and land use classes. This suggests that wild pigs forage widely into grassland floodplains, savanna woodlands and coastal shrublands when conditions permit. Our regional model showed that rainforests are a key year-round habitat for feral pig breeding (Fig 7). This was partly due to model assumptions. For example, as actual freshwater presence was inadequately mapped in densely vegetated rainforests, we assumed that water was uniformly available (Table 1). Site-scale studies have already provided a more detailed understanding of the fine-grained variations in actual habitat use within this broadly suitable habitat type [18,20–22].

While all parts of northern Australia contained at least some suitable wild pig habitat, our model suggests that wild pigs are less widely distributed in the region than previously thought. For example, moderately suitable habitat in Western Australia totalled between 3000 km^2^ (dry) and 32,000 km^2^ (wet) while Cowled *et al*.’s [32] model predicted 89,000 km^2^ of suitable habitat in the same area. Similarly, West [24] reported that wild pigs are widespread throughout Queensland while our model found that only 16% (dry) to 54% (wet) of this area contained all required resources for persistent wild pig breeding. We note that (a) such area estimates are highly dependent on habitat thresholds and therefore are unreliable and difficult to compare between studies, and (b) our figures are possibly overestimates–they refer to a threshold of *HSI* ≥ 40, yet validation plots (Fig 5) suggested that a higher, more restrictive threshold (e.g. *HSI* ≥ 60) may better discriminate between suitable and unsuitable habitat in most environments. We suggest our lower estimates are defensible when considering a number of methodological improvements in our study. First, we specifically modelled resource requirements of wild pig breeding herds, which are more limiting than those of solitary boars [14,17,21]. Previous statistical models [32] and mapping studies [24,70] did not make this distinction, although long-term occupancy critically relies on breeding. Second, previous work did not distinguish between seasonal scenarios but included any location where wild pigs have occurred or may occur. This may have approximated wet season habitat and likely led to overestimations by failing to consider the dry season as a limiting factor for wild pigs in Australia’s north [16,19]. Finally, we used a finer spatial resolution (1 ha) than previous studies (25 km^2^ in [32] or ~250 km^2^ in [24]), resulting in more detailed predictions and less upscaling error (e.g. one “suitable” pixel equalled 25 km^2^ in [32], even if only a fraction of this area actually contained suitable habitat).

### Model evaluation and validation

Model results were robust to both expert opinion and a range of independently collected validation data sets of wild pig presence. All six experts contributing to the final consensus model provided similar parameter estimates for the Bayesian network model of habitat suitability. That is, they all agreed that water and food resources are more important to persistent wild pig breeding in northern Australia than protection from heat and protection from disturbance. However, validation revealed inconsistent performance between expert models in some instances. We attributed this model uncertainty mainly to different expert assumptions about wild pigs’ resource-seeking home range movements, described in elicited response-to-pattern curves (Fig 3). Our simple approach to reducing uncertainty, equal-weighted expert averaging [52,58], yielded similar, or more accurate, results than the best expert models for most validation data.

While the averaged habitat suitability model always performed well, there were differences between seasonal scenarios and validation data. This points to some limitations of our study. First, most validation data were collected in environments with an above-average proportion of suitable wild pig habitat. Second, model parameters were elicited from experts with field knowledge mostly from resource-abundant environments in the eastern study region and applied to all of northern Australia. Model accuracy in resource-poor inland environments and in those portions of the study region for which no validation data was available and that were outside the expertise of our panel of experts (especially Western Australia) needs further investigation [74]. For example, wild pigs may also sustain themselves in ‘unsuitable’ habitat during the dry season from resources not included in our model (e.g. carrion and other animal matter). Third, reporting bias in the data points used for validation [75] and our definition of validation backgrounds may have affected validation results. For example, most data sets were biased towards highly suitable sites as management-focussed survey efforts mostly concentrated on sites known to be impacted by wild pigs. Less suitable sites, which nevertheless support wild pig breeding, may therefore have few or no data points recorded, resulting in somewhat inflated *CBI* values. Further, different buffering choices for defining the ALA, NAQS or Balkanu validation backgrounds may have yielded poorer (likely for backgrounds that are more narrowly defined around presence records than our 15 km buffer) or enhanced (likely for larger backgrounds that encompass high proportions of unsuitable habitat especially in the spatially disjunct ALA data set, S1 Fig) performance results. Fourth, the *Continuous Boyce Index* validation method is well suited to presence-only validation data but cannot evaluate model specificity, i.e. its ability to correctly predict absences and minimize false positives [64,67]. Finally, we applied the same expert-elicited mobility thresholds and distance-dependent response-to-pattern curves to the entire study region and both seasonal scenarios. Yet, wild pigs may adjust their resource-seeking home range movements to environmental conditions [17,21,73] and in fact respond to aspects of landscape structure other than distance such as resource composition or heterogeneity [76].

A listing of study limitations is most useful for guiding future research. We refer to van Klinken *et al*. [42] who suggest methods to evaluate possible errors in expert-elicited models. In particular, our study may be usefully improved by: (1) systematically collecting presence/ absence data, also from resource-poor environments, to eliminate reporting bias in validation data and enable evaluation of both model sensitivity and specificity; (2) field-validating the accuracy of spatial data proxies and whether they match the states of model explanatory variables; and (3) revising model parameters with experts from the Northern Territory and Western Australia; and conducting a multi-scale study [59,77] to better understand wild pigs’ response to spatial patterns of resources with varying quality in different types of environments.

### Management implications

Our regional-scale, seasonally-specific and rigorously validated results can serve to better manage the impacts of wild pigs in northern Australia. For example, we estimated habitat suitability per broad vegetation and land use types. When combined with information on impacts or costs in each category [12,29,78], this may help to more accurately quantify environmental or economic impacts across any area of interest. Habitat suitability could also be analysed in other management units to justify investments in population control or, if verified by stratified field surveys, to serve as a monitoring baseline in adaptive management programs. Further, because habitat suitability was explicitly referenced to wild pig breeding herds, it is a useful indicator of establishment risk for infectious animal diseases, which often depend on a persistent supply of young susceptible animals [27,28]. Habitat connectivity for wild pigs is also a critical parameter in understanding disease spread, but has not been explicitly studied in northern Australia. Future models may integrate validation results from this study to derive habitat quality thresholds for patch delineation [67] and inverse habitat suitability to define resistance to movement, i.e. the cost of traversing habitat of different quality [79]. Finally, our resource-based model captures the relative importance of four habitat requirements as well as spatial interactions such as landscape complementation or supplementation [80]. This knowledge may be used to manipulate resource access at strategic locations (e.g. by exclusion fencing or local eradication) and model the effect on habitat suitability and connectivity within the broader landscape.

## Supporting information

S1 Tables Bayesian network conditional probability tables to model resource quality indices for water, food, protection from heat stress and protection from disturbance as well as an index of habitat suitability for wild pig breeding (S2 Fig). (XLSX)

S2 Tables Bayesian network model variables with definitions (S2.1 Table) and spatial data proxies with methods for reclassifying categorical attributes to match the states of model explanatory variables (S2.2-S2.6 Tables). (DOCX)

S1 Appendix Rcode spatial pattern suitability analysis. (PDF)

S2 Appendix Rcode validation. (PDF)

S1 Fig Maps of seasonal habitat suitability per validation area. (DOCX)

S2 Fig Seasonal habitat suitability per state and per broad vegetation type. (DOCX)
