# Supplementary material for: Modelling seasonal habitat suitability for wide-ranging species: Invasive wild pigs in northern Australia
Source: PLoS One. 2017 May 4;12(5):e0177018. doi: 10.1371/journal.pone.0177018 (PMC5417638; doi:10.1371/journal.pone.0177018)
Supplement: S2 Tables — (DOCX) [file pone.0177018.s002.docx]

**S2.1 Table** Bayesian network model variables and their states, with definitions.

| Node & definition | State | (interval) | State definition |
| --- | --- | --- | --- |
| - Habitat suitability index (*HSI*) - Whether feral pig mobs are able to breed and persist | Very high | (80-100) | Feral pig mobs always able to breed, strong population growth |
|  | High | (60-80) | ... usually able to breed, population growth |
|  | Moderate | (40-60) | ... occasionally able to breed, population maintenance |
|  | Low | (20-40) | ... usually unable to breed, population decline |
|  | Very low | (0-20) | … always unable to breed, strong population decline |
| - Water suitability index (*SI_Water_*) - Whether there is sufficient potable water available to meet drinking requirements of feral pig mobs | Very good | (80-100) | Drinking water fully sufficient to meet requirements |
|  | Good | (60-80) | ... usually sufficient to meet requirements |
|  | Moderate | (40-60) | ... occasionally sufficient to meet requirements |
|  | Poor | (20-40) | ... usually limiting breeding and persistence |
|  | Very poor | (0-20) | … strongly limiting breeding and persistence |
| - Water quality index (*x_Water_*) - Presence of accessible sources of potable water | Very good | (80-100) | Potable water present and fully accessible |
|  | Good | (60-80) | ... present and usually accessible |
|  | Moderate | (40-60) | ... present and accessible with some difficulty |
|  | Poor | (20-40) | ... present and usually inaccessible |
|  | Very poor | (0-20) | … absent or fully inaccessible |
| - Freshwater presence - Presence of potable water | Yes | (50-100) | Potable water present |
|  | No | (0-50) | Water absent or non-potable (salty/brackish) |
| - Terrain ruggedness - Accessibility of water source due to terrain ruggedness | Low | (67-100) | Level and/or low-lying terrain |
|  | Moderate | (33-67) | Moderately rugged terrain |
|  | High | (0-33) | Highly rugged terrain |
| - Food suitability index (*SI_Food_*) - Whether there are sufficient food resources available to meet nutritional and energy requirements of breeding sows | Very good | (80-100) | Food resources fully sufficient to meet requirements |
|  | Good | (60-80) | ... usually sufficient to meet requirements |
|  | Moderate | (40-60) | ... occasionally sufficient to meet requirements |
|  | Poor | (20-40) | ... usually limiting breeding and persistence |
|  | Very poor | (0-20) | … strongly limiting breeding and persistence |
| - Food quality index (*x_Food_*) - Presence and nutritional value of food resources | Very good | (80-100) | Food resources present and of very high nutritional value |
|  | Good | (60-80) | ... present and of high nutritional value |
|  | Moderate | (40-60) | ... present and of some nutritional value |
|  | Poor | (20-40) | ... present and of low nutritional value |
|  | Very poor | (0-20) | … absent or of very low nutritional value |
| - Food quality - Quality (protein content) and accessibility of food resources | High | (67-100) | High quality food resources and readily accessible |
|  | Moderate | (33-67) | Moderate quality food resources and/or restricted accessibility |
|  | Low | (0-33) | Low quality food resources or inaccessible |
| - Food quantity - Quantity (energy content) of food resources | High | (67-100) | High quantity (energy-rich) of food resources |
|  | Moderate | (33-67) | Moderate quantity (some energy) of food resources |
|  | Low | (0-33) | Low quantity (little or no energy) of food resources |

| - Heat protection suitability index (*SI_Heat_*) - Whether there is sufficient heat refuge available to meet protection requirements of feral pig mobs | Very good | (80-100) | Heat refuge fully sufficient to meet requirements |
| --- | --- | --- | --- |
|  | Good | (60-80) | ... usually sufficient to meet requirements |
|  | Moderate | (40-60) | ... occasionally sufficient to meet requirements |
|  | Poor | (20-40) | ... usually limiting breeding and persistence |
|  | Very poor | (0-20) | … strongly limiting breeding and persistence |
| - Heat protection quality index (*x_Heat_*) - Presence and quality of refuge from heat stress conditions | Very good | (80-100) | Insignificant heat stress or refuge offers full protection |
|  | Good | (60-80) | Refuge offers good protection from heat stress conditions |
|  | Moderate | (40-60) | Refuge offers some protection ... |
|  | Poor | (20-40) | Refuge offers little protection ... |
|  | Very poor | (0-20) | Refuge offers no or very little protection ... |
| - Heat stress - Heat stress conditions from daytime temperatures | Low | (67-100) | Insignificant heat stress |
|  | Moderate | (33-67) | Some heat stress from prolonged moderate daytime temperatures |
|  | High | (0-33) | Significant heat stress from prolonged high daytime temperatures |
| - Shady vegetation cover - Cool microclimate provided by shady vegetation canopy | Good | (67-100) | Deep shading provided by a dense vegetation canopy |
|  | Moderate | (33-67) | Dappled shading provided by an open vegetation canopy |
|  | Poor | (0-33) | No or little shading provided by a very sparse vegetation canopy |
| - Disturbance protection suitability index (*SI_Disturbance_*) - Whether there is sufficient disturbance refuge available to meet protection requirements of feral pig mobs | Very good | (80-100) | Disturbance refuge fully sufficient to meet requirements |
|  | Good | (60-80) | ... usually sufficient to meet requirements |
|  | Moderate | (40-60) | ... occasionally sufficient to meet requirements |
|  | Poor | (20-40) | ... usually limiting breeding and persistence |
|  | Very poor | (0-20) | … strongly limiting breeding and persistence |
| - Disturbance protection quality index (*x_Disturbance_*) - Presence and quality of refuge from disturbance stress | Very good | (80-100) | Insignificant disturbance stress or refuge offers full protection |
|  | Good | (60-80) | Refuge offers good protection from disturbance stress |
|  | Moderate | (40-60) | Refuge offers some protection ... |
|  | Poor | (20-40) | Refuge offers little protection ... |
|  | Very poor | (0-20) | Refuge offers no or very little protection ... |
| - Dense vegetation cover - Cover provided by understory vegetation | Good | (67-100) | Good cover provided by dense understory vegetation |
|  | Moderate | (33-67) | Moderate cover provided by open or medium-height vegetation |
|  | Poor | (0-33) | No or little cover provided by sparse or tall vegetation |
| - Disturbance stress - Disturbance stress from human or predator interference | Low | (67-100) | Insignificant disturbance stress |
|  | Moderate | (33-67) | Some disturbance stress |
|  | High | (0-33) | Significant disturbance stress |
| - Intensity of control | Low | (50-100) | No or low/moderate impact control activities |
|  | High | (0-50) | High impact control activities |
| - Frequency of control | Low | (50-100) | No or infrequent/occasional control activities |
|  | High | (0-50) | Sustained control effort (throughout and across years) |
| - Predator presence | Low | (50-100) | No or insignificant numbers of predators are present |
|  | High | (0-50) | Significant numbers of predators are present |

The first state listed is considered most favourable to wild pig breeding. Numerical intervals corresponding to each state are also listed in brackets.

**S2.2 Table** Spatial data proxies linked to model explanatory variables and methods for reclassifying data attributes into state-specific categories.

| Node [seasonal?] | Spatial layer | | |  | Reclassification into state-specific categories [name of state] | | | | | |
| --- | --- | --- | --- | --- | --- | --- | --- | --- | --- | --- |
|  | Name | Date, file type | Source, reference |  | Rationale, method |  | Best | | Middle | Worst |
| Freshwater presence [seasonal] | Queensland wetland data V.3 - wetland areas / points (springs) | 2012, ESRI shapefile (1:100,000) | Dept. of Environment & Heritage Protection QLD, EPA 2005 |  | Classified areas as ‘perennial/ephemeral/none’ from Wetclass & WtrRegime attributes, excluded non-potable water from Salinmod attribute, classified points as ‘perennial’ except if dormant |  | - dry: perennial - wet: perennial + ephemeral - [Yes] | | n/a | - dry: none + ephemeral - wet: none - [No] |
|  | Geofabric Surface Cartography V.2.1 – HydroPoint / Dam | 2012, FDGB feature class (1:250,000) | Bureau of Meteorology, BOM 2012 |  | Classified as ‘perennial’ except abandoned/dry or saline |  | - dry/wet: perennial - [Yes] | | n/a | - dry/wet: none - [No] |
|  | Geofabric Surface Cartography V.2.1 – HydroArea / WaterBody | 2012, FDGB feature class (1:250,000) | Bureau of Meteorology, BOM 2012 |  | Classified HydroAreas as ‘ephemeral/none’ and WaterBody as ‘perennial/ephemeral/none’ from AHGFType, Perennial & SrcFType attributes |  | - dry: perennial - wet: perennial + ephemeral - [Yes] | | n/a | - dry: none + ephemeral - wet: none - [No] |
|  | Present Major Vegetation Subgroups (MVS), NVIS V.4.1 | 2012, FDGB grid (100m) | Federal Dept. of the Environment, DEWR 2007 |  | Water bodies inadequately mapped in tropical, sub-tropical or warm temperate rainforest, classified as water present (non-limiting) |  | - MVS 2 & 6 - [Yes] | | n/a | - MVS other - [No] |
| Terrain ruggedness | 3 sec SRTM derived Digital Elevation Model, V.1.0 | 2010, FDGB grid (3 arcsec) | Geoscience Australia, Gallant et al. 2011 |  | Calculated terrain ruggedness index (TRI) using gdaldem algorithm (based on Wilson et al. 2007), classified visually as thresholds scale dependent |  | - TRI < 12 - [Low] | | - TRI 12-24 - [Moderate] | - TRI >24 - [High] |
| Food quality [seasonal] | Present Major Vegetation Subgroups (MVS), NVIS V.4.1 | 2012, FDGB grid (100m) | Federal Dept. of the Environment, DEWR 2007 |  | Estimated food quality in natural systems under good / poor accessibility of below-ground protein, elicited from experts & Murray et al. 2015 |  | Classification see  S2.3 Table | | | |
|  | Catchment scale Land Use of Australia (CLUM) | 2015, FDGB grid (50m) | Federal Dept. of Agriculture, ABARES 2011 |  | Estimated food quality in modified systems (land use class) under good / poor accessibility of below-ground protein, elicited from experts & Murray et al. 2015 |  | Classification see  S2.4 Table | | | |
|  | Monthly relative soil moisture upper layer (WRel1), historical run 26j / operational v26 | 2015, ESRI float (0.05°) | CSIRO, Raupach et al. 2009 |  | Calculated average WRel1 for each scenario, classified visually into good / poor accessibility of below-ground protein sources |  | - WRel1 > 20 - [Good] | | n/a | - WRel1 ≤ 20 - [Poor] |
| Food quantity [seasonal] | Monthly fractional cover of Photosynthetic Vegetation (f_PV_), V.3.0.1 | 2015, TIFF grid (500m) | Office of Environment & Heritage NSW, Gill et al. 2014 |  | Proxy indicator of vegetative productivity/ overall energy, calculated average f_PV_ for each scenario, equal interval classes from Guerschman pers. comm. |  | - f_PV_ >66 - [High] | | - f_PV_ 33-66 - [Moderate] | - f_PV_ <33 - [Low] |
| Heat stress [seasonal] | Monthly mean maximum temperature (T_max_) | 2015, ASCII grid (0.05°) | Bureau of Meteorology, Jones et al. 2009 |  | Calculated average T_max_ for each scenario, class thresholds elicited from experts |  | - < 29 °C - [Low] | | - 29-31 °C - [Moderate] | - > 31 °C - [High] |
| Shady vegetation cover | Persistent Green Vegetation Fraction 2000 - 2010 (PGVF), V.2.0 | 2012, TIFF grid (30m) | Joint Remote Sensing Research Program, Johansen et al. 2012 |  | Proxy indicator of woody vegetation foliage cover, class thresholds from Johansen pers. comm., NCST 2009 & DSITIA 2012 |  | - PGVF > 140 - [Good] | - PGVF 121-140 - [Moderate] | | - PGVF <121 - [Poor] |
| Dense vegetation cover | Present Major Vegetation Groups (MVG), NVIS V.4.1 | 2012, FDGB grid (100m) | Federal Dept. of the Environment, DEWR 2007 |  | Estimated cover in natural land systems (secondary land use class), elicited from experts & Murray et al. 2015 |  | Classification see  S2.5 Table | | | |
|  | Catchment scale Land Use of Australia (CLUM) | 2015, FDGB grid (50m) | Federal Dept. of Agriculture, ABARES 2011 |  | Estimated cover in modified land systems (land use class), elicited from experts & Murray et al. 2015 |  | Classification see  S2.6 Table | | | |

References:

ABARES / Australian Bureau of Agricultural and Resource Economics and Sciences (2011). *Guidelines for land use mapping in Australia: principles, procedures and definitions, fourth edition*. Commonwealth of Australia, Canberra.

BOM / Bureau of Meteorology (2012). *Australian hydrological geospatial fabric (geofabric) data product specification: surface cartography Version 2.1*. Bureau of Meteorology, Canberra.

DEWR / Department of the Environment and Water Resources (2007). *Australia’s native vegetation: a summary of Australia’s major vegetation groups, 2007*. Commonwealth of Australia, Canberra.

DSITIA / Department of Science, IT, Innovation and the Arts (2012). *Land cover change in Queensland 2009–10: a Statewide Landcover and Trees Study (SLATS) report*. The State of Queensland, Brisbane.

EPA / Environmental Protection Agency (2005). *Wetland mapping and classification methodology. Overall framework: a method to provide baseline mapping and classification for wetlands in Queensland, version 1.2*. The State of Queensland, Brisbane.

Gallant, JC, Dowling, TI, Read, AM, Wilson, N, Tickle, P et al. (2011). *1 second SRTM derived products user guide, Version 1.0.4*. Geoscience Australia, Canberra.

Gill, T, Heidenreich, S & Guerschman, JP (2014). *MODIS monthly fractional cover: product creation and distribution*. Joint Remote Sensing Research Program Publication Series.

Johansen, K, Gill, T, Trevithick, R, Armston, J, Scarth, P et al. (2012). "Validation of Landsat based time-series of persistent green-vegetation fraction for Australia." *Proceedings of the XXII Congress of the International Photogrammetry and Remote Sensing Society 2012*. Melbourne.

Jones, DA, Wang, W & Fawcett, R (2009). "High-quality spatial climate data-sets for Australia." *Australian Meteorological and Oceanographic Journal* 58(4): 233-248.

Murray, J, Froese, J, Perry, J, Navarro Garcia, J & van Klinken, R (2015). *Impact modelling for rabbits and feral pigs in the QMDB*. CSIRO Biosecurity, Brisbane.

NCST / National Committee on Soil and Terrain (2009). *Australian soil and land survey field handbook*. CSIRO Publishing, Collingwood.

Raupach, M, Briggs, P, Haverd, V, King, E, Paget, M et al. (2009). *Australian Water Availability Project (AWAP), CSIRO Marine and Atmospheric Research component: final report for phase 3*. Technical Report No. 013. Centre for Australian Weather and Climate Research (Bureau of Meteorology and CSIRO), Melbourne.

Wilson, MFJ, O’Connell, B, Brown, C, Guinan, JC & Grehan, AJ (2007). "Multiscale terrain analysis of multibeam bathymetry data for habitat mapping on the continental slope." *Marine Geodesy* 30(1-2): 3-35.

**S2.3 Table** Classification of *Present Major Vegetation Subgroups* (MVS V.4.1 *) into food quality under good and poor accessibility of below-ground protein sources.

| MVS No. | MVS Name | Type | Food quality (access) | |
| --- | --- | --- | --- | --- |
|  |  |  | (good) | (poor) |
| 2 | Tropical or sub-tropical rainforest | Rainforest | High | High |
| 4 | Eucalyptus open forests, shrubby understorey | Woodland | Low | Low |
| 5 | Eucalyptus open forests, grassy understorey | Woodland | Moderate | Low |
| 6 | Warm Temperate Rainforest | Rainforest | Moderate | Moderate |
| 7 | Tropical Eucalyptus forest & woodlands, tall annual grassy understorey | Woodland | Low | Low |
| 8 | Eucalyptus woodlands, shrubby understorey | Woodland | Low | Low |
| 9 | Eucalyptus woodlands, tussock grass understorey | Woodland | Low | Low |
| 10 | Eucalyptus woodlands, hummock grass understorey | Woodland | Low | Low |
| 11 | Tropical mixed spp forests & woodlands | Woodland | Moderate | Low |
| 12 | Callitris forests & woodlands | Woodland | Low | Low |
| 13 | Brigalow forests & woodlands | Woodland | Low | Low |
| 14 | Other Acacia forests & woodlands | Woodland | Low | Low |
| 15 | Melaleuca open forests & woodlands | Woodland | Moderate | Low |
| 16 | Other forests & woodlands | Woodland | Low | Low |
| 18 | Eucalyptus low open woodlands with hummock grass | Woodland | Low | Low |
| 19 | Eucalyptus low open woodlands with tussock grass | Woodland | Moderate | Low |
| 20 | Mulga woodlands +/- tussock grass +/- forbs | Shrubland | Low | Low |
| 21 | Other Acacia tall open shrublands & [tall] shrublands | Shrubland | Low | Low |
| 23 | Acacia open woodlands & shrublands with hummock grass | Shrubland | Low | Low |
| 24 | Acacia open woodlands & shrublands +/- tussock grass | Shrubland | Low | Low |
| 25 | Acacia open woodlands & sparse shrublands, shrubby understorey | Shrubland | Low | Low |
| 26 | Casuarina & Allocasuarina forests & woodlands | Woodland | Low | Low |
| 27 | Mallee with hummock grass | Shrubland | Low | Low |
| 28 | Low closed forest or tall closed shrublands | Shrubland | Low | Low |
| 30 | Heath | Shrubland | Low | Low |
| 31 | Saltbush & Bluebush shrublands | Shrubland | Low | Low |
| 32 | Other shrublands | Shrubland | Low | Low |
| 33 | Hummock grasslands | Grassland | Low | Low |
| 34 | Mitchell grass tussock grasslands | Grassland | Moderate | Low |
| 35 | Blue grass & tall bunch grass tussock grasslands | Grassland | High | Low |
| 37 | Other tussock grasslands | Grassland | Moderate | Low |
| 38 | Wet tussock grassland with herbs, sedges or rushes, herblands or ferns | Wetland | High | High |
| 39 | Mixed chenopod, samphire +/- forbs | Shrubland | Low | Low |
| 40 | Mangroves | Mangroves | High | High |
| 41 | Saline or brackish sedgelands or grasslands | Wetland | High | High |
| 42 | Naturally bare, sand, rock, claypan, mudflat | None | Low | Low |
| 43 | Salt lakes & lagoons | Wetland | Moderate | Moderate |
| 44 | Freshwater, dams, lakes, lagoons or aquatic plants | Wetland | Moderate | Moderate |
| 45 | Mulga open woodlands & sparse shrublands +/- tussock grass | Shrubland | Low | Low |
| 46 | Sea, estuaries (includes seagrass) | None | Low | Low |
| 47 | Eucalyptus open woodlands with shrubby understorey | Woodland | Low | Low |
| 48 | Eucalyptus open woodlands, grassy understorey | Woodland | Moderate | Low |
| 49 | Melaleuca shrublands & open shrublands | Shrubland | Low | Low |
| 51 | Mulga woodlands & shrublands with hummock grass | Shrubland | Low | Low |
| 52 | Mulga open woodlands & sparse shrublands with hummock grass | Shrubland | Low | Low |
| 53 | Eucalyptus low open woodlands, shrubby understorey | Woodland | Low | Low |
| 56 | Eucalyptus open woodlands, chenopod or samphire understorey | Woodland | Low | Low |
| 57 | Lignum shrublands & wetlands | Wetland | Moderate | Moderate |
| 59 | Eucalyptus woodlands, ferns, herbs, sedges, rushes, wet tussock grasses | Woodland | High | Moderate |
| 60 | Eucalyptus open forests, ferns, herbs, sedges, rushes, wet tussock grasses | Woodland | High | Moderate |
| 62 | Dry rainforest or vine thickets | Rainforest | Moderate | Low |
| 63 | Sedgelands, rushs or reeds | Wetland | High | High |
| 64 | Other grasslands | Grassland | Moderate | Low |
| 70 | Callitris open woodlands | Woodland | Low | Low |
| 71 | Casuarina & Allocasuarina open woodlands, tussock grass understorey | Woodland | Low | Low |
| 74 | Casuarina & Allocasuarina open woodlands, shrubby understorey | Woodland | Low | Low |
| 75 | Melaleuca open woodlands | Woodland | Moderate | Low |
| 79 | Other open Woodlands | Woodland | Low | Low |
| 80 | Other sparse shrublands & sparse heathlands | Shrubland | Low | Low |
| 90 | Regrowth or modified forests & woodlands | Unknown | Low | Low |
| 92 | Regrowth or modified graminoids | Unknown | Low | Low |
| 96 | Unclassified Forest | Woodland | Low | Low |
| 97 | Unclassified native vegetation | Unknown | Low | Low |
| 98 | Cleared, non-native vegetation, buildings | None | Low | Low |
| 99 | Unknown/No data | Unknown | Low | Low |

* Reference: Department of the Environment and Water Resources (2007). *Australia’s native vegetation: a summary of Australia’s major vegetation groups, 2007*. Commonwealth of Australia, Canberra.

**S2.4 Table** Classification of *Australian Land Use and Management* (ALUM V.7 *) primary (and secondary/ tertiary where applicable) classes into food quality under good and poor accessibility of below-ground protein sources.

| Primary (secondary, tertiary) ALUM class | Food quality (access) | |
| --- | --- | --- |
|  | (good) | (poor) |
| 1 Conservation and natural environments | Low | Low |
| 2 Production from relatively natural environments | Low | Low |
| 3 Production from dryland agriculture and plantations  3.1 Plantation forestry, 3.6 Land in transition | Low | Low |
| 3 Production from dryland agriculture and plantations  3.2 Grazing modified pastures | Moderate | Low |
| 3 Production from dryland agriculture and plantations  3.3 Cropping | High | Moderate |
| 3 Production from dryland agriculture and plantations  3.4 Perennial horticulture, 3.5 Seasonal horticulture | High | High |
| 4 Production from irrigated agriculture and plantations  4.1 Irrigated plantation forestry, 4.5.5 Irrigated turf farming & 4.6 Irrigated land in transition | Low | Low |
| 4 Production from irrigated agriculture and plantations  4.2 Grazing irrigated modified pastures | Moderate | Moderate |
| 4 Production from irrigated agriculture and plantations  4.3 Irrigated cropping, 4.4 Irrigated perennial horticulture, 4.5 Irrigated seasonal horticulture (except 4.5.5) | High | High |
| 5 Intensive uses | Low | Low |
| 6 Water (except 6.3.0 & 6.3.1) | Low | Low |
| 6 Water  6.3.0 River, 6.3.1 River - conservation | Moderate | Moderate |

* Reference: Australian Bureau of Agricultural and Resource Economics and Sciences (2011). *Guidelines for land use mapping in Australia: principles, procedures and definitions, fourth edition*. Commonwealth of Australia, Canberra.

**S2.5 Table** Classification of *Present Major Vegetation Groups* (MVG V.4.1 *) into dense vegetation cover.

| MVG No. | MVG Name | Type | Cover |
| --- | --- | --- | --- |
| 1 | Rainforests and Vine Thickets | Rainforest | Good |
| 2 | Eucalypt Tall Open Forests | Woodland | Moderate |
| 3 | Eucalypt Open Forests | Woodland | Moderate |
| 4 | Eucalypt Low Open Forests | Woodland | Moderate |
| 5 | Eucalypt Woodlands | Woodland | Moderate |
| 6 | Acacia Forests and Woodlands | Woodland | Moderate |
| 7 | Callitris Forests and Woodlands | Woodland | Moderate |
| 8 | Casuarina Forests and Woodlands | Woodland | Moderate |
| 9 | Melaleuca Forests and Woodlands | Woodland | Moderate |
| 10 | Other Forests and Woodlands | Woodland | Moderate |
| 11 | Eucalypt Open Woodlands | Woodland | Poor |
| 12 | Tropical Eucalypt Woodlands/Grasslands | Woodland | Moderate |
| 13 | Acacia Open Woodlands | Shrubland | Poor |
| 14 | Mallee Woodlands and Shrublands | Shrubland | Moderate |
| 15 | Low Closed Forests and Tall Closed Shrublands | Shrubland | Good |
| 16 | Acacia Shrublands | Shrubland | Moderate |
| 17 | Other Shrublands | Shrubland | Moderate |
| 18 | Heathlands | Shrubland | Good |
| 19 | Tussock Grasslands | Grassland | Poor |
| 20 | Hummock Grasslands | Grassland | Poor |
| 21 | Other Grasslands, Herblands, Sedgelands and Rushlands | Wetland | Poor |
| 22 | Chenopod Shrublands, Samphire Shrublands and Forblands | Shrubland | Poor |
| 23 | Mangroves | Mangroves | Good |
| 24 | Inland aquatic - freshwater, salt lakes, lagoons | Wetland | Poor |
| 25 | Cleared, non-native vegetation, buildings | None/modified | Poor |
| 26 | Unclassified native vegetation | None/modified | Poor |
| 27 | Naturally bare - sand, rock, claypan, mudflat | None/modified | Poor |
| 28 | Sea and estuaries | None/modified | Poor |
| 29 | Regrowth, modified native vegetation | None/modified | Poor |
| 30 | Unclassified Forest | Woodland | Moderate |
| 31 | Other Open Woodlands | Woodland | Poor |
| 32 | Mallee Open Woodlands and Sparse Mallee Shrublands | Shrubland | Poor |
| 99 | Unknown/no data | None/modified | Poor |

* Reference: Department of the Environment and Water Resources (2007). *Australia’s native vegetation: a summary of Australia’s major vegetation groups, 2007*. Commonwealth of Australia, Canberra.

**S2.6 Table** Classification of *Australian Land Use and Management* (ALUM V.7 *) primary (and secondary/ tertiary where applicable) classes into dense vegetation cover.

| Primary (secondary, tertiary) ALUM class | Cover |
| --- | --- |
| 1 Conservation and natural environments | Poor |
| 2 Production from relatively natural environments  2.1 Grazing natural vegetation | Poor |
| 2 Production from relatively natural environments  2.2 Production forestry | Moderate |
| 3 Production from dryland agriculture and plantations  3.1 Plantation forestry, 3.3.5 Sugar, 3.4 Perennial horticulture (except 3.4.0) | Moderate |
| 3 Production from dryland agriculture and plantations  3.2 Grazing modified pastures, 3.3 Cropping (except 3.3.5), 3.4.0 Perennial horticulture, 3.5 Seasonal horticulture, 3.6 Land in transition | Poor |
| 4 Production from irrigated agriculture and plantations  4.1 Irrigated plantation forestry, 4.3.5 Irrigated sugar, 4.4 Irrigated perennial horticulture (except 4.4.0 & 4.4.7), 4.5.1 Irrigated seasonal fruits | Moderate |
| 4 Production from irrigated agriculture and plantations  4.2 Grazing irrigated modified pastures, 4.3 Irrigated cropping (except 4.3.5), 4.4.0 Irrigated perennial horticulture, 4.4.7 Irrigated perennial vegetables & herbs, 4.5 Irrigated seasonal horticulture (except 4.5.1), 4.6 Irrigated land in transition | Poor |
| 5 Intensive uses | Poor |
| 6 Water | Poor |

* Reference: Australian Bureau of Agricultural and Resource Economics and Sciences (2011). *Guidelines for land use mapping in Australia: principles, procedures and definitions, fourth edition*. Commonwealth of Australia, Canberra.
