# Supplementary material for: Modelling seasonal habitat suitability for wide-ranging species: Invasive wild pigs in northern Australia
Source: PLoS One. 2017 May 4;12(5):e0177018. doi: 10.1371/journal.pone.0177018 (PMC5417638; doi:10.1371/journal.pone.0177018)
Supplement: S1 Appendix — (PDF) [file pone.0177018.s003.pdf]

S1 Appendix. Rcode spatial pattern suitability analysis (PDF)

Jens G. Froese

25 April 2017

This document provides a detailed, reproducible description of the spatial pattern suitability analysis methodology. It is Supporting Information (S1 Appendix) to the manuscript:

> Froese JG, Smith CS, Durr PA, McAlpine CA, van Klinken RD. Modelling seasonal habitat suitability for wide-ranging species: invasive wild pigs in northern Australia. PLoS ONE.

It is written in `R Markdown` ([1]) and `knitr` ([2]), two `R` ([3]) packages for writing dynamic, reproducible reports. A `.zip` file containing data inputs to reproduce analyses can be downloaded from Dryad (`http://dx.doi.org/10.5061/dryad.v103v`). Some parts of the code used to print this document have been surpressed to enhance readability. A generalized version of the code is available at URL `https://github.com/jgfroese/PATTSI` .

Load required R packages

R package `raster` ([4]) and its dependencies are required for spatial pattern suitability analysis. Session information incl. package versions are listed at the bottom of this document.

```
require(raster) # for all analyses of raster objects incl. moving window analysis
```

---

1. Expert elicitation

For each habitat variable (i.e. its modelled resource quality indices `xr`), we elicited a distance-dependent response-to-pattern curve (`fDr`) from each individual expert (see manuscript Fig 3). These curves followed a step-wise pattern, because we discretised both:

- distance into five equal distance bands (“very close”, “close”, “medium”, “far” and “very far”), relative to each expert’s defined mobility threshold (i.e. 1km, 2km or 3km).
- resource suitability indices (`SIr`) into five equal classes (“very good (80-100)”, “good (60-80)”, “moderate (40-60)”, “poor (20-40)” and “very poor (0-20)”), see manuscript S2.1 Table.

We asked experts to relate each distance band to a corresponding suitability class under the assumption that other variables do not constrain suitability. To fill two elicitation gaps (expert 3 did not define `fDDisturbance` and expert 6 did not define `fDHeat`) we

- applied all other experts’ `fDr` to the missing expert’s defined mobility threshold (Expert3 = 2km, Expert6 = 3km)
- computed the average `fDr` and used it for the missing expert’s model

To derive distance weights for computation of **2. Resource suitability indices, Step 4**, we used the mid-points of elicited suitability index classes divided by 100 (e.g. class “moderate (40-60)” = `SIr` 50 = weight 0.5). For class “very good (80-100)” we did not use the mid-point `SIr = 90` but assigned `SIr = 100` (= weight 1.0) to avoid unintended distance penalties (i.e. an adjacent resource of quality `xr = 60` should compute as distance-weighted suitability `SIr = 60` (if weight is 1.0) and not `SIr = 54` (if weight is 0.9)).

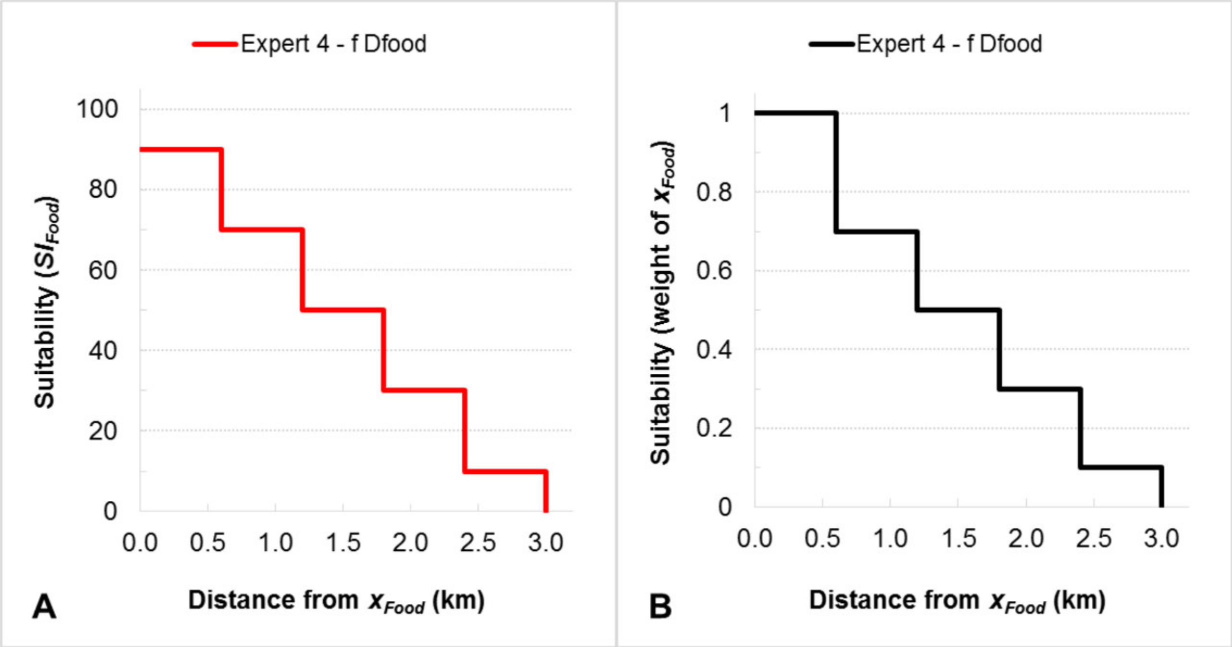

Figure 1 Step-wise distance-dependent response-to-pattern curve for habitat variable “food” elicited from expert 4 (A) and distance weights derived for computation of food suitability indices (B).

---

2. Resource suitability indices

Goal

Focal pixel resource suitability index (`SIr`) depends on the distance of a (numerical) habitat variable.

Method:

- Generate a circular moving window where each position is weighted by its distance from the focal pixel (radius/weights derived from **1. Expert elicitation**).
- compute the focal pixel `SIr` as the highest weighted value (`xr`) of a habitat variable within this moving window.

Step 1

Define a function that returns a circular matrix of given radius and resolution and assigns value `1` if matrix position <= radius and value `NA` if matrix position > radius (Source: [5]).

```
make_circ_filter <- function(radius, res){
  circ_filter <- matrix(NA, nrow=1+(2*radius/res), ncol=1+(2*radius/res))
  dimnames(circ_filter)[[1]] <- seq(-radius, radius, by=res)
  dimnames(circ_filter)[[2]] <- seq(-radius, radius, by=res)
  sweeper <- function(mat){
    for(row in 1:nrow(mat)){
      for(col in 1:ncol(mat)){
        dist <- sqrt((as.numeric(dimnames(mat)[[1]])[row])^2 +
                     (as.numeric(dimnames(mat)[[1]])[col])^2)
        if(dist<=radius) {mat[row, col]<-1}
      }
    }
    return(mat)
  }
  out <- sweeper(circ_filter)
  return(out)
}
```

Step 2

Apply function to generate five matrices with different radii (= distance bands), relative to each expert’s defined mobility threshold (i.e. 1km for Expert1, 2km for Experts 3/5 and 3km for Experts 2/4/6).

```
res <- 1 # resolution (= pixel size, e.g. 100m)
mr.1 <- 10 # matrix radius (= mobility threshold, must be multiple of res, e.g 1km = 10 x 10
0m)
m.1 <- make_circ_filter(mr.1, res)
m.2 <- make_circ_filter((mr.1/5)*4, res)
m.3 <- make_circ_filter((mr.1/5)*3, res)
m.4 <- make_circ_filter((mr.1/5)*2, res)
m.5 <- make_circ_filter((mr.1/5), res)
```

Replace `value==1` with unique temp value in ascending order from largest to smallest matrix.

```
m.1[m.1 == 1] <- 1
m.2[m.2 == 1] <- 2
m.3[m.3 == 1] <- 3
m.4[m.4 == 1] <- 4
m.5[m.5 == 1] <- 5
```

Step 3

Combine the five matrices into one (two at a time starting with the smallest):

```
a.5 <- array(NA, dim(m.4), dimnames(m.4)) # create temp array of size = larger matrix
a.5[rownames(m.5), colnames(m.5)] <- m.5 # ... with values = smaller matrix
m.4 <- pmax(m.4, a.5, na.rm = TRUE) # combine values: larger matrix + temp array
a.4 <- array(NA, dim(m.3), dimnames(m.3)) # repeat with: output + next-larger matrix
a.4[rownames(m.4), colnames(m.4)] <- m.4
m.3 <- pmax(m.3, a.4, na.rm = TRUE)
a.3 <- array(NA, dim(m.2), dimnames(m.2))
a.3[rownames(m.3), colnames(m.3)] <- m.3
m.2 <- pmax(m.2, a.3, na.rm = TRUE)
a.2 <- array(NA, dim(m.1), dimnames(m.1))
a.2[rownames(m.2), colnames(m.2)] <- m.2
m.band.1 <- pmax(m.1, a.2, na.rm = TRUE)
```

```
##     -10 -9 -8 -7 -6 -5 -4 -3 -2 -1 0  1  2  3  4  5  6  7  8  9 10
## -10  NA NA NA NA NA NA NA NA NA NA 1 NA NA NA NA NA NA NA NA NA NA
## -9   NA NA NA NA NA NA  1  1  1  1 1  1  1  1  1 NA NA NA NA NA NA
## -8   NA NA NA NA  1  1  1  1  1  1 2  1  1  1  1  1  1 NA NA NA NA
## -7   NA NA NA  1  1  1  1  2  2  2 2  2  2  2  1  1  1  1 NA NA NA
## -6   NA NA  1  1  1  2  2  2  2  2 3  2  2  2  2  2  1  1  1 NA NA
## -5   NA NA  1  1  2  2  2  3  3  3 3  3  3  3  2  2  2  1  1 NA NA
## -4   NA  1  1  1  2  2  3  3  3  3 4  3  3  3  3  2  2  1  1  1 NA
## -3   NA  1  1  2  2  3  3  3  4  4 4  4  4  3  3  3  2  2  1  1 NA
## -2   NA  1  1  2  2  3  3  4  4  4 5  4  4  4  3  3  2  2  1  1 NA
## -1   NA  1  1  2  2  3  3  4  4  5 5  5  4  4  3  3  2  2  1  1 NA
## 0     1  1  2  2  3  3  4  4  5  5 5  5  5  4  4  3  3  2  2  1  1
## 1    NA  1  1  2  2  3  3  4  4  5 5  5  4  4  3  3  2  2  1  1 NA
## 2    NA  1  1  2  2  3  3  4  4  4 5  4  4  4  3  3  2  2  1  1 NA
## 3    NA  1  1  2  2  3  3  3  4  4 4  4  4  3  3  3  2  2  1  1 NA
## 4    NA  1  1  1  2  2  3  3  3  3 4  3  3  3  3  2  2  1  1  1 NA
## 5    NA NA  1  1  2  2  2  3  3  3 3  3  3  3  2  2  2  1  1 NA NA
## 6    NA NA  1  1  1  2  2  2  2  2 3  2  2  2  2  2  1  1  1 NA NA
## 7    NA NA NA  1  1  1  1  2  2  2 2  2  2  2  1  1  1  1 NA NA NA
## 8    NA NA NA NA  1  1  1  1  1  1 2  1  1  1  1  1  1 NA NA NA NA
## 9    NA NA NA NA NA NA  1  1  1  1 1  1  1  1  1 NA NA NA NA NA NA
## 10   NA NA NA NA NA NA NA NA NA NA 1 NA NA NA NA NA NA NA NA NA NA
```

Repeat steps 2 and 3 for mobility thresholds 2km and 3km.

```
mr.2 <- 20 # moving window radius 2km (= 20 x 100m)
mr.3 <- 30 # moving window radius 3km (= 30 x 100m)
...
m.band.2
m.band.3
```

Step 4

Replace temp values with expert-elicited weight for each distance band (`SIdata.zip` file containing `f_DFood.csv` and other weights derived from **1. Expert elicitation** can be downloaded from Dryad (`http://dx.doi.org/10.5061/dryad.v103v`)).

```
csv.Food = read.csv("SIdata/S3Appendix/fD/f_DFood.csv")
csv.Food.E1 <- subset(csv.Food, Expert == 'Expert1') # for Expert1 use m.band.1
m.band.1[m.band.1 == 1] <- csv.Food.E1$X1
m.band.1[m.band.1 == 2] <- csv.Food.E1$X0.8
m.band.1[m.band.1 == 3] <- csv.Food.E1$X0.6
m.band.1[m.band.1 == 4] <- csv.Food.E1$X0.4
m.band.1[m.band.1 == 5] <- csv.Food.E1$X0.2
m.band.Food.E1 <- m.band.1
csv.Food.E2 <- subset(csv.Food, Expert == 'Expert2') # for Expert2 use m.band.3
m.band.3[m.band.3 == 1] <- csv.Food.E2$X3
m.band.3[m.band.3 == 2] <- csv.Food.E2$X2.4
m.band.3[m.band.3 == 3] <- csv.Food.E2$X1.8
m.band.3[m.band.3 == 4] <- csv.Food.E2$X1.2
m.band.3[m.band.3 == 5] <- csv.Food.E2$X0.6
m.band.Food.E2 <- m.band.3
csv.Food.E3 <- subset(csv.Food, Expert == 'Expert3') # for Expert3 use m.band.2
m.band.2[m.band.2 == 1] <- csv.Food.E3$X2
m.band.2[m.band.2 == 2] <- csv.Food.E3$X1.6
m.band.2[m.band.2 == 3] <- csv.Food.E3$X1.2
m.band.2[m.band.2 == 4] <- csv.Food.E3$X0.8
m.band.2[m.band.2 == 5] <- csv.Food.E3$X0.4
m.band.Food.E3 <- m.band.2
```

Step 5

Perform moving window analysis using function `focal {raster}` with parameters (`SIdata.zip` file containing raster layers with resource quality indices `xr` can be downloaded from Dryad (`http://dx.doi.org/10.5061/dryad.v103v`)).

```
r = raster("SIdata/S3Appendix/GIS/Food-quality-dry.tif") # raster layer with numerical resou
rce quality index, e.g. Food quality in dry season scenario
w = m.band.Food.E1 # moving window is banded weights matrix, e.g. Expert 1 f_Dfood
fun = max # focal pixel takes highest weighted resource quality index within moving window
```

WARNING! The following process may take several hours depending on the size of r and w.

```
r.f <- focal(r, w, fun, na.rm = TRUE, pad = FALSE, padValue = NA) # na.rm = TRUE ignores NoD
ata
r.m <- mask(r.f, r) # extract by r to remove padded edges
writeRaster(r.m, filename = paste("SIdata/S3Appendix/out/Food-SI-dry_E1.tif", sep="")) # sav
e ouput raster
```

Repeat steps 4 and 5 for all four habitat variables and six experts in two seasonal scenarios:

- Water wet/dry = 12 runs
- Food wet/dry = 12 runs
- Heat wet/dry = 12 runs
- Disturbance global scenario = 6 runs

References

[1] Allaire, J.J. et al. 2016. Package ‘rmarkdown’: dynamic documents for R. URL `http://rmarkdown.rstudio.com/` .

[2] Xie, Y. 2016. Package ‘knitr’: a general-purpose package for dynamic report generation in R. URL `http://yihui.name/knitr/` .

[3] RCoreTeam 2015. R: a language and environment for statistical computing. R Foundation for Statistical Computing, Vienna, Austria. URL `http://www.R-project.org/` .

[4] Hijmans, R.J. 2015. Package ‘raster’: geographic data analysis and modeling. URL `http://cran.r-project.org/web/packages/raster/` .

[5] Scroggie, M. 2012. Applying a circular moving window filter to raster data in R. URL `https://scrogster.wordpress.com/2012/10/05/applying-a-circular-moving-window-filter-to-raster-data-i`

Session information

```
## Session info -------------------------------------------------------------
```

```
##  setting  value
##  version  R version 3.1.3 (2015-03-09)
##  system   x86_64, mingw32
##  ui       RTerm
##  language (EN)
##  collate  English_Australia.1252
##  tz       Australia/Brisbane
##  date     2017-04-25
```

```
## Packages -----------------------------------------------------------------
```

```
##  package   * version date       source
##  devtools    1.10.0  2016-01-23 CRAN (R 3.1.3)
##  digest      0.6.8   2014-12-31 CRAN (R 3.1.3)
##  evaluate    0.8     2015-09-18 CRAN (R 3.1.3)
##  formatR     1.2.1   2015-09-18 CRAN (R 3.1.3)
##  htmltools   0.3     2015-12-29 CRAN (R 3.1.3)
##  knitr       1.12.3  2016-01-22 CRAN (R 3.1.3)
##  lattice     0.20-30 2015-02-22 CRAN (R 3.1.3)
##  magrittr    1.5     2014-11-22 CRAN (R 3.1.3)
##  memoise     1.0.0   2016-01-29 CRAN (R 3.1.3)
##  raster    * 2.4-20  2015-09-08 CRAN (R 3.1.3)
##  Rcpp        0.12.1  2015-09-10 CRAN (R 3.1.3)
##  rgdal       1.1-1   2015-11-02 CRAN (R 3.1.3)
##  rmarkdown   0.9.2   2016-01-01 CRAN (R 3.1.3)
##  sp        * 1.2-1   2015-10-18 CRAN (R 3.1.3)
##  stringi     1.0-1   2015-10-22 CRAN (R 3.1.3)
##  stringr     1.0.0   2015-04-30 CRAN (R 3.1.3)
##  yaml        2.1.13  2014-06-12 CRAN (R 3.1.3)
```
