# Supplementary material for: Modelling seasonal habitat suitability for wide-ranging species: Invasive wild pigs in northern Australia
Source: PLoS One. 2017 May 4;12(5):e0177018. doi: 10.1371/journal.pone.0177018 (PMC5417638; doi:10.1371/journal.pone.0177018)
Supplement: S2 Appendix — (PDF) [file pone.0177018.s004.pdf]

S2 Appendix. Rcode validation (PDF)

Jens G. Froese

25 April 2017

This document provides a detailed, reproducible description of the methodology used to evaluate and validate performance of habitat suitability models using the *Continuous Boyce Index (CBI)*. It is Supporting Information (S2 Appendix) to the manuscript:

> Froese JG, Smith CS, Durr PA, McAlpine CA, van Klinken RD. Modelling seasonal habitat suitability for wide-ranging species: invasive wild pigs in northern Australia. PLoS ONE.

The document is written in `R Markdown` ([1]) and `knitr` ([2]), two `R` ([3]) packages for writing dynamic, reproducible reports. A `.zip` file containing data inputs to reproduce analyses can be downloaded from Dryad (`http://dx.doi.org/10.5061/dryad.v103v`). Some parts of the code used to print this document have been suppressed to enhance readability. A generalized version of the code is available at URL `https://github.com/jgfroese/HSI-CBI-validation` .

Load required R packages

R packages `ecospat` ([4]), `data.table` ([5]) and `zoo` ([6]) and their dependencies are required for *HSI*-*CBI*-validation. Session information incl. package versions are listed at the bottom of this document.

```
require(data.table) # for function `setnames`
require(ecospat) # for function `ecospat.boyce`
require(zoo) # for plotting function `na.fill`
```

---

1. Data preparation

Step 1

This R script requires two `.TXT` files for each model / validation data combination, which have to be prepared in package `{raster}` or alternative GIS software as described below. These files can be downloaded from Dryad (`http://dx.doi.org/10.5061/dryad.v103v`).

1. Expected *HSI* across validation background:
    - define validation backgrounds (`Balkanu = BLKW` , `Lakefield = LKNP` , `Oyala Thumotang = OTNP` , `NAQS` , `ALA` - see manuscript **Table 2**)
    - mask raster layers of individual expert models and an average model by each validation background
    - export raster attribute tables to `.TXT` with 3 columns: [ID], [HSI], [pixel count]
2. Predicted *HSI* at wild pig presence records:
    - convert presence records of each validation data set into raster layer
    - combine masked raster layer of each expert/average model with each set of presence records
    - export raster attribute tables to `.TXT` with 5 columns: [ID], [Value], [pixel count], [HSI], [number of presence records per pixel]

Step 2

Compute model-predicted *HSI* at wild pig presence records.

First, read `.TXT` files for each model (Expert1, Expert2, …, averaged) / validation presences (BLKW, LKNP, OTNP, NAQS, ALA) combination as data frame,

```
pred.E1.wetBLKW = read.csv("SIdata/S4Appendix/wet/Predicted/SiPred_wet_E1_BLKW1000.txt")
...
pred.E6.wetBLKW = read.csv("SIdata/S4Appendix/wet/Predicted/SiPred_wet_E6_BLKW1000.txt")
pred.av.wetBLKW = read.csv("SIdata/S4Appendix/wet/Predicted/SiPred_wet_Eall_BLKW1000.txt")
pred.E1.dryBLKW = read.csv("SIdata/S4Appendix/dry/Predicted/SiPred_dry_E1_BLKW1000.txt")
...
pred.E6.dryBLKW = read.csv("SIdata/S4Appendix/dry/Predicted/SiPred_dry_E6_BLKW1000.txt")
pred.av.dryBLKW = read.csv("SIdata/S4Appendix/dry/Predicted/SiPred_dry_Eall_BLKW1000.txt")
...
pred.av.wetLKNP = read.csv("SIdata/S4Appendix/wet/Predicted/SiPred_wet_Eall_LKNP1000.txt")
...
pred.av.dryLKNP = read.csv("SIdata/S4Appendix/dry/Predicted/SiPred_dry_Eall_LKNP1000.txt")
...
pred.av.wetOTNP = read.csv("SIdata/S4Appendix/wet/Predicted/SiPred_wet_Eall_OTNP1000.txt")
...
pred.av.dryNAQS = read.csv("SIdata/S4Appendix/dry/Predicted/SiPred_dry_Eall_NAQS1000.txt")
...
pred.av.wetALA = read.csv("SIdata/S4Appendix/wet/Predicted/SiPred_wet_Eall_ALA1000.txt")
...
pred.av.dryALA = read.csv("SIdata/S4Appendix/dry/Predicted/SiPred_dry_Eall_ALA1000.txt")
```

and combine all data frames in a list for faster analysis.

```
pred.list <- list (pred.E1.wetBLKW, pred.E2.wetBLKW, pred.E3.wetBLKW, pred.E4.wetBLKW, pred.E5.wetBLKW,
pred.E6.wetBLKW, pred.av.wetBLKW, pred.E1.dryBLKW, pred.E2.dryBLKW, pred.E3.dryBLKW, pred.E4.dryBLKW, p
red.E5.dryBLKW, pred.E6.dryBLKW, pred.av.dryBLKW, pred.E1.wetLKNP, pred.E2.wetLKNP, pred.E3.wetLKNP, pr
ed.E4.wetLKNP, pred.E5.wetLKNP, pred.E6.wetLKNP, pred.av.wetLKNP, pred.E1.dryLKNP, pred.E2.dryLKNP, pre
d.E3.dryLKNP, pred.E4.dryLKNP, pred.E5.dryLKNP, pred.E6.dryLKNP, pred.av.dryLKNP,  pred.E1.wetOTNP, pre
d.E2.wetOTNP, pred.E3.wetOTNP, pred.E4.wetOTNP,pred.E5.wetOTNP, pred.E6.wetOTNP, pred.av.wetOTNP, pred.
E1.dryNAQS, pred.E2.dryNAQS, pred.E3.dryNAQS, pred.E4.dryNAQS, pred.E5.dryNAQS, pred.E6.dryNAQS, pred.a
v.dryNAQS, pred.E1.wetALA, pred.E2.wetALA, pred.E3.wetALA, pred.E4.wetALA, pred.E5.wetALA, pred.E6.wetA
LA, pred.av.wetALA, pred.E1.dryALA, pred.E2.dryALA, pred.E3.dryALA, pred.E4.dryALA, pred.E5.dryALA, pre
d.E6.dryALA, pred.av.dryALA)
n.list <- 56 # the number of data frames in your list
```

Then, homogenise the five column names for all data frames,

```
for (i in seq_along(pred.list)) {
  setnames(pred.list[[i]], c("ID", "Value", "Pixelcount", "HSI", "Presences"))
}
```

and calculate the total number of presence records per *HSI* value (one pixel may contain multiple records).

```
pred.sum.list <- vector("list", n.list)
for (i in seq_along(pred.list)) {
  pred.sum.list[[i]] <- aggregate(cbind(Pixelcount*Presences)~HSI, data = pred.list[[i]], sum)
}
```

Finally, add descriptive column names to the new list of data frames,

```
for (i in seq_along(pred.sum.list)) {
  setnames(pred.sum.list[[i]], c("HSI", "Presences"))
}
```

and convert it into a list of vectors (= *HSI* at feral pig presence records),

```
pred.v.list = vector("list", n.list)
for (i in seq_along(pred.sum.list)) {
  pred.v = vector()
  for (j in 1:length(pred.sum.list[[i]]$Presences)) {
    for (k in 1:pred.sum.list[[i]][j, 2]) {
      pred.v <-append(pred.v, pred.sum.list[[i]][j, 1])
    }
  }
  pred.v.list[[i]] <- append(pred.v.list[[i]], pred.v)
}
```

e.g. *HSI* of model 1 at Balkanu presence records (first in list).

Step 3

Compute model-predicted *HSI* across validation backgrounds.

First, read `.TXT` files for each model (Expert1, Expert2, …, averaged) / validation background (BLKW, LKNP, OTNP, NAQS, ALA) combination as data frame,

```
exp.E1.wetBLKW = read.csv("SIdata/S4Appendix/wet/Expected/SiExp_wet_E1_BLKW1000.txt")
...
exp.E6.wetBLKW = read.csv("SIdata/S4Appendix/wet/Expected/SiExp_wet_E6_BLKW1000.txt")
exp.av.wetBLKW = read.csv("SIdata/S4Appendix/wet/Expected/SiExp_wet_Eall_BLKW1000.txt")
exp.E1.dryBLKW = read.csv("SIdata/S4Appendix/dry/Expected/SiExp_dry_E1_BLKW1000.txt")
...
exp.E6.dryBLKW = read.csv("SIdata/S4Appendix/dry/Expected/SiExp_dry_E6_BLKW1000.txt")
exp.av.dryBLKW = read.csv("SIdata/S4Appendix/dry/Expected/SiExp_dry_Eall_BLKW1000.txt")
...
exp.av.wetLKNP = read.csv("SIdata/S4Appendix/wet/Expected/SiExp_wet_Eall_LKNP1000.txt")
...
exp.av.dryLKNP = read.csv("SIdata/S4Appendix/dry/Expected/SiExp_dry_Eall_LKNP1000.txt")
...
exp.av.wetOTNP = read.csv("SIdata/S4Appendix/wet/Expected/SiExp_wet_Eall_OTNP1000.txt")
...
exp.av.dryNAQS = read.csv("SIdata/S4Appendix/dry/Expected/SiExp_dry_Eall_NAQS1000.txt")
...
exp.av.wetALA = read.csv("SIdata/S4Appendix/wet/Expected/SiPred_wet_Eall_ALA1000.txt")
...
exp.av.dryALA = read.csv("SIdata/S4Appendix/dry/Expected/SiPred_dry_Eall_ALA1000.txt")
```

and combine all data frames in a list for faster analysis.

```
exp.list <- list (exp.E1.wetBLKW, exp.E2.wetBLKW, exp.E3.wetBLKW, exp.E4.wetBLKW, exp.E5.wetBLKW, exp.E6.wetBLKW, exp.av.wetBLKW, exp.E1.dryBLKW, exp.E2.dryBLKW, exp.E3.dryBLKW, exp.E4.dryBLKW, exp.E5.dryBLKW, exp.E6.dryBLKW, exp.av.dryBLKW, exp.E1.wetLKNP, exp.E2.wetLKNP, exp.E3.wetLKNP, exp.E4.wetLKNP, exp.E5.wetLKNP, exp.E6.wetLKNP, exp.av.wetLKNP, exp.E1.dryLKNP, exp.E2.dryLKNP, exp.E3.dryLKNP, exp.E4.dryLKNP, exp.E5.dryLKNP, exp.E6.dryLKNP, exp.av.dryLKNP, exp.E1.wetOTNP, exp.E2.wetOTNP, exp.E3.wetOTNP, exp.E4.wetOTNP, exp.E5.wetOTNP, exp.E6.wetOTNP, exp.av.wetOTNP, exp.E1.dryNAQS, exp.E2.dryNAQS, exp.E3.dryNAQS, exp.E4.dryNAQS, exp.E5.dryNAQS, exp.E6.dryNAQS, exp.av.dryNAQS, exp.E1.wetALA, exp.E2.wetALA, exp.E3.wetALA, exp.E4.wetALA, exp.E5.wetALA, exp.E6.wetALA, exp.av.wetALA, exp.E1.dryALA, exp.E2.dryALA, exp.E3.dryALA, exp.E4.dryALA, exp.E5.dryALA, exp.E6.dryALA, exp.av.dryALA)
```

Then, homogenise the three column names for all data frames,

```
for (i in seq_along(exp.list)) {
  setnames(exp.list[[i]], c("ID", "HSI", "Pixelcount"))
}
```

and convert it into a list of vectors (= *HSI* across validation backgrounds [potentially very large - see manuscript **Table 1**]).

```
exp.v.list = vector("list", n.list)
for (i in seq_along(exp.list)) {
  exp.v = vector()
  for (j in 1:length(exp.list[[i]]$Pixelcount)) {
    for (k in 1:exp.list[[i]][j, 3]) {
      exp.v <-append(exp.v, exp.list[[i]][j, 2])
    }
  }
  exp.v.list[[i]] <- append(exp.v.list[[i]], exp.v)
}
```

---

2. Data analysis

Step 4

Apply function `boyce {ecospat}` to each model (Expert1, Expert2, …, averaged) / validation data (BLKW, LKNP, OTNP, NAQS, ALA) combination with parameters:

```
exp.v.list[[i]] # Expected HSI across background ([[1]] = E1.wetBLKW, ... , [[56]] = av.dryALA)
pred.v.list[[i]] # Predicted HSI at presences ([[1]] = E1.wetBLKW, ... , [[56]] = av.dryALA)
```

```
nclass = 0 # defaults to moving window (continuous, classification-independent) computation with argume
nts:
  window.w = 10 # moving window width (i.e. 10 adjacent HSI values are considered in each computation)
  res = 100 # resolution factor (i.e. 100 computations across the total range of HSI)
  PEplot = F # no PEplot is generated (customised plots - see manuscript Figure 6)
```

```
boyce.E1.wetBLKW <- ecospat.boyce(exp.v.list[[1]], pred.v.list[[1]], nclass, window.w, res, PEplot)
...
boyce.E6.wetBLKW <- ecospat.boyce(exp.v.list[[6]], pred.v.list[[6]], nclass, window.w, res, PEplot)
boyce.av.wetBLKW <- ecospat.boyce(exp.v.list[[7]], pred.v.list[[7]], nclass, window.w, res, PEplot)
boyce.E1.dryBLKW <- ecospat.boyce(exp.v.list[[8]], pred.v.list[[8]], nclass, window.w, res, PEplot)
...
boyce.E6.dryBLKW <- ecospat.boyce(exp.v.list[[13]], pred.v.list[[13]], nclass, window.w, res, PEplot)
boyce.av.dryBLKW <- ecospat.boyce(exp.v.list[[14]], pred.v.list[[14]], nclass, window.w, res, PEplot)
...
boyce.av.wetLKNP <- ecospat.boyce(exp.v.list[[21]], pred.v.list[[21]], nclass, window.w, res, PEplot)
...
boyce.av.dryLKNP <- ecospat.boyce(exp.v.list[[28]], pred.v.list[[28]], nclass, window.w, res, PEplot)
...
boyce.av.wetOTNP <- ecospat.boyce(exp.v.list[[35]], pred.v.list[[35]], nclass, window.w, res, PEplot)
...
boyce.av.dryNAQS <- ecospat.boyce(exp.v.list[[42]], pred.v.list[[42]], nclass, window.w, res, PEplot)
...
boyce.av.wetALA <- ecospat.boyce(exp.v.list[[49]], pred.v.list[[49]], nclass, window.w, res, PEplot)
...
boyce.av.dryALA <- ecospat.boyce(exp.v.list[[56]], pred.v.list[[56]], nclass, window.w, res, PEplot)
```

Step 5

Investigate results of *CBI* analysis.

Combine all results in list for faster analysis,

```
boyce.list <- list (boyce.E1.wetBLKW, boyce.E2.wetBLKW, boyce.E3.wetBLKW, boyce.E4.wetBLKW, boyce.E5.we
tBLKW, boyce.E6.wetBLKW, boyce.av.wetBLKW, boyce.E1.dryBLKW, boyce.E2.dryBLKW, boyce.E3.dryBLKW, boyce.
E4.dryBLKW, boyce.E5.dryBLKW, boyce.E6.dryBLKW, boyce.av.dryBLKW, boyce.E1.wetLKNP, boyce.E2.wetLKNP, b
oyce.E3.wetLKNP, boyce.E4.wetLKNP, boyce.E5.wetLKNP, boyce.E6.wetLKNP, boyce.av.wetLKNP, boyce.E1.dryLK
NP, boyce.E2.dryLKNP, boyce.E3.dryLKNP, boyce.E4.dryLKNP, boyce.E5.dryLKNP, boyce.E6.dryLKNP, boyce.av.
dryLKNP, boyce.E1.wetOTNP, boyce.E2.wetOTNP, boyce.E3.wetOTNP, boyce.E4.wetOTNP, boyce.E5.wetOTNP, boyc
e.E6.wetOTNP, boyce.av.wetOTNP, boyce.E1.dryNAQS, boyce.E2.dryNAQS, boyce.E3.dryNAQS, boyce.E4.dryNAQS,
boyce.E5.dryNAQS, boyce.E6.dryNAQS, boyce.av.dryNAQS, boyce.E1.wetALA, boyce.E2.wetALA, boyce.E3.wetALA
, boyce.E4.wetALA, boyce.E5.wetALA, boyce.E6.wetALA, boyce.av.wetALA, boyce.E1.dryALA, boyce.E2.dryALA,
boyce.E3.dryALA, boyce.E4.dryALA, boyce.E5.dryALA, boyce.E6.dryALA, boyce.av.dryALA)
```

and print *CBI* (`$Spearman.cor`) for all model / validation data combinations (see manuscript **Table 3**),

```
CBI.list = vector("list", n.list)
for (i in seq_along(boyce.list)) {
  CBI.list[[i]] <- append(CBI.list[[i]], round(boyce.list[[i]]$Spearman.cor, digits = 2))
}
CBI.list
```

e.g. *CBI* of expert model 1 validated against wet season Balkanu presence records (first in list).

Step 6

Compute proportion of validation background expected to be highly or very highly suitable habitat (*HSI* >= 60) for all model / validation data combinations (see manuscript **Table 3**),

```
t.HSI <- 59.99 # HSI threshold
HSI.60 <- vector("list", n.list)
for (i in seq_along(HSI.60)) {
  HSI.60[[i]] <- aggregate(Pixelcount~HSI > t.HSI, data = exp.list[[i]], sum) / sum(exp.list[[i]]$Pixel
count)
}
HSI.60.list <- vector("list", n.list)
for (i in seq_along(HSI.60.list)) {
  HSI.60.list[[i]] <- append(HSI.60.list[[i]], (round(HSI.60[[i]][2, "Pixelcount"] * 100, digits = 0)))
}
HSI.60.list
```

e.g. *HSI* 60 of expert model 1 in wet season Balkanu validation background (first in list).

Step 7

Compare *P/E ratio* between individual expert models and an averaged model for each validation data set.

Create a nested list (first list expert/average models per validation data set, then list validation data sets),

```
boyce.wetBLKW <- list (boyce.E1.wetBLKW, boyce.E2.wetBLKW, boyce.E3.wetBLKW, boyce.E4.wetBLKW, boyce.E5
.wetBLKW, boyce.E6.wetBLKW, boyce.av.wetBLKW)
boyce.dryBLKW <- list (boyce.E1.dryBLKW, boyce.E2.dryBLKW, boyce.E3.dryBLKW, boyce.E4.dryBLKW, boyce.E5
.dryBLKW, boyce.E6.dryBLKW, boyce.av.dryBLKW)
boyce.wetLKNP <- list (boyce.E1.wetLKNP, boyce.E2.wetLKNP, boyce.E3.wetLKNP, boyce.E4.wetLKNP, boyce.E5
.wetLKNP, boyce.E6.wetLKNP, boyce.av.wetLKNP)
boyce.dryLKNP <- list (boyce.E1.dryLKNP, boyce.E2.dryLKNP, boyce.E3.dryLKNP, boyce.E4.dryLKNP, boyce.E5
.dryLKNP, boyce.E6.dryLKNP, boyce.av.dryLKNP)
boyce.wetOTNP <- list (boyce.E1.wetOTNP, boyce.E2.wetOTNP, boyce.E3.wetOTNP, boyce.E4.wetOTNP, boyce.E5
.wetOTNP, boyce.E6.wetOTNP, boyce.av.wetOTNP)
boyce.dryNAQS <- list (boyce.E1.dryNAQS, boyce.E2.dryNAQS, boyce.E3.dryNAQS, boyce.E4.dryNAQS, boyce.E5
.dryNAQS, boyce.E6.dryNAQS, boyce.av.dryNAQS)
boyce.wetALA <- list (boyce.E1.wetALA, boyce.E2.wetALA, boyce.E3.wetALA, boyce.E4.wetALA, boyce.E5.wetA
LA, boyce.E6.wetALA, boyce.av.wetALA)
boyce.dryALA <- list (boyce.E1.dryALA, boyce.E2.dryALA, boyce.E3.dryALA, boyce.E4.dryALA, boyce.E5.dryA
LA, boyce.E6.dryALA, boyce.av.dryALA)
plot.v.list <- list ("Balkanu (wet season)" = boyce.wetBLKW, "Balkanu (dry season)" = boyce.dryBLKW, "L
akefield (wet season)" = boyce.wetLKNP, "Lakefield (dry season)" = boyce.dryLKNP, "Oyala Thumotang (wet
season)" = boyce.wetOTNP,  "NAQS (dry season)" = boyce.dryNAQS, "ALA NT (wet season)" = boyce.wetALA, "
ALA NT (dry season)" = boyce.dryALA)
```

and plot from nested list (see manuscript **Fig 5**).

```
graphics.off()
par(mfrow = c(4, 4), mar = c(2, 2, 3, 0), oma = c(4, 3, 0, 0))
for (i in seq_along(plot.v.list)) {
  plot(plot.v.list[[i]][[1]]$HS, plot.v.list[[i]][[1]]$F.ratio, type = "n", xlab = '', ylab = '',
       xlim = c(15, 80), ylim = c(0, 3), main = paste(names(plot.v.list[i])))
  lines(plot.v.list[[i]][[1]]$HS, na.fill(plot.v.list[[i]][[1]]$F.ratio, 0), col = "black", lty = 1)
  lines(plot.v.list[[i]][[2]]$HS, na.fill(plot.v.list[[i]][[2]]$F.ratio, 0), col = "black", lty = 2)
  lines(plot.v.list[[i]][[3]]$HS, na.fill(plot.v.list[[i]][[3]]$F.ratio, 0), col = "black", lty = 3)
  lines(plot.v.list[[i]][[4]]$HS, na.fill(plot.v.list[[i]][[4]]$F.ratio, 0), col = "black", lty = 4)
  lines(plot.v.list[[i]][[5]]$HS, na.fill(plot.v.list[[i]][[5]]$F.ratio, 0), col = "black", lty = 5)
  lines(plot.v.list[[i]][[6]]$HS, na.fill(plot.v.list[[i]][[6]]$F.ratio, 0), col = "black", lty = 6)
  lines(plot.v.list[[i]][[7]]$HS, na.fill(plot.v.list[[i]][[7]]$F.ratio, 0), col = "red", lty = 1, lwd
= 2)
  mtext(LETTERS[i], side = 3, line = -2, adj = 0.05)
  plot.new()
  legend("left", c((paste("Expert 1, CBI =", round(plot.v.list[[i]][[1]]$Spearman.cor, digits = 2))),
                   (paste("Expert 2, CBI =", round(plot.v.list[[i]][[2]]$Spearman.cor, digits = 2))),
                   (paste("Expert 3, CBI =", round(plot.v.list[[i]][[3]]$Spearman.cor, digits = 2))),
                   (paste("Expert 4, CBI =", round(plot.v.list[[i]][[4]]$Spearman.cor, digits = 2))),
                   (paste("Expert 5, CBI =", round(plot.v.list[[i]][[5]]$Spearman.cor, digits = 2))),
                   (paste("Expert 6, CBI =", round(plot.v.list[[i]][[6]]$Spearman.cor, digits = 2))),
                   (paste("Averaged, CBI =", round(plot.v.list[[i]][[7]]$Spearman.cor, digits = 2))))
,
         bty = "n", lty = c(1,2,3,4,5,6,1), col = c("black", "black", "black", "black", "black", "black
", "red"), lwd = c(1, 1, 1, 1, 1, 1, 2), y.intersp = 1.3, title = expression(bold(Legend)), title.adj =
0.05)
}
mtext(expression(italic(Habitat~suitability~index~(HSI))), side = 1, outer = TRUE, cex = 1.2, line = 2.
2)
mtext(expression(italic(Predicted-to-expected~(P/E)~ratio)), side = 2, outer = TRUE, cex = 1.2, line =
0.8)
```

References

[1] Allaire, J.J. et al. 2016. Package ‘rmarkdown’: dynamic documents for R. URL `http://rmarkdown.rstudio.com/` .

[2] Xie, Y. 2016. Package ‘knitr’: a general-purpose package for dynamic report generation in R. URL `http://yihui.name/knitr/` .

[3] RCoreTeam 2015. R: a language and environment for statistical computing. R Foundation for Statistical Computing, Vienna, Austria. URL `http://www.R-project.org/` .

[4] Broennimann, O. 2015. Package ‘ecospat’: spatial ecology miscellaneous methods. URL `http://cran.r-project.org/web/packages/ecospat/` .

[5] Dowle, M. et al. 2015. Package ‘data.table’: extension of data.frame. URL `https://github.com/Rdatatable/data.table/wiki/` .

[6] Zeileis, A. et al. 2015. Package ‘zoo’: S3 infrastructure for regular and irregular time series. URL `http://zoo.R-Forge.R-project.org/` .

Session information

```
## Session info -------------------------------------------------------------
```

```
##  setting  value
##  version  R version 3.1.3 (2015-03-09)
##  system   x86_64, mingw32
##  ui       RTerm
##  language (EN)
##  collate  English_Australia.1252
##  tz       Australia/Brisbane
##  date     2017-04-25
```

```
## Packages -----------------------------------------------------------------
```

```
##  package      * version date       source
##  abind          1.4-3   2015-03-13 CRAN (R 3.1.3)
##  acepack        1.3-3.3 2013-05-03 CRAN (R 3.1.3)
##  ade4         * 1.7-2   2015-04-14 CRAN (R 3.1.3)
##  adehabitatHR   0.4.14  2015-07-22 CRAN (R 3.1.3)
##  adehabitatLT   0.3.20  2015-07-22 CRAN (R 3.1.3)
##  adehabitatMA   0.3.10  2015-07-22 CRAN (R 3.1.3)
##  ape          * 3.3     2015-05-29 CRAN (R 3.1.3)
##  biomod2        3.1-64  2014-12-10 CRAN (R 3.1.3)
##  boot           1.3-15  2015-02-24 CRAN (R 3.1.3)
##  chron          2.3-47  2015-06-24 CRAN (R 3.1.3)
##  CircStats      0.2-4   2012-10-29 CRAN (R 3.1.3)
##  class          7.3-12  2015-02-11 CRAN (R 3.1.3)
##  cluster        2.0.1   2015-01-31 CRAN (R 3.1.3)
##  codetools      0.2-10  2015-01-17 CRAN (R 3.1.3)
##  colorspace     1.2-6   2015-03-11 CRAN (R 3.1.3)
##  data.table   * 1.9.6   2015-09-19 CRAN (R 3.1.3)
##  deldir         0.1-9   2015-03-09 CRAN (R 3.1.3)
##  devtools       1.10.0  2016-01-23 CRAN (R 3.1.3)
##  digest         0.6.8   2014-12-31 CRAN (R 3.1.3)
##  dismo          1.0-12  2015-03-15 CRAN (R 3.1.3)
##  ecodist        1.2.9   2013-12-03 CRAN (R 3.1.3)
##  ecospat      * 1.1     2015-03-06 CRAN (R 3.1.3)
##  evaluate       0.8     2015-09-18 CRAN (R 3.1.3)
##  foreach      * 1.4.3   2015-10-13 CRAN (R 3.1.3)
##  foreign        0.8-63  2015-02-20 CRAN (R 3.1.3)
##  formatR        1.2.1   2015-09-18 CRAN (R 3.1.3)
##  Formula        1.2-1   2015-04-07 CRAN (R 3.1.3)
##  gam          * 1.12    2015-05-13 CRAN (R 3.1.3)
##  gbm          * 2.1.1   2015-03-11 CRAN (R 3.1.3)
##  ggplot2        2.1.0   2016-03-01 CRAN (R 3.1.3)
##  goftest        1.0-3   2015-07-03 CRAN (R 3.1.3)
##  gridExtra      2.0.0   2015-07-14 CRAN (R 3.1.3)
##  gtable         0.1.2   2012-12-05 CRAN (R 3.1.3)
##  hexbin         1.27.1  2015-08-19 CRAN (R 3.1.3)
##  Hmisc          3.17-0  2015-09-21 CRAN (R 3.1.3)
##  htmltools      0.3     2015-12-29 CRAN (R 3.1.3)
##  iterators      1.0.8   2015-10-13 CRAN (R 3.1.3)
##  knitr          1.12.3  2016-01-22 CRAN (R 3.1.3)
##  lattice      * 0.20-30 2015-02-22 CRAN (R 3.1.3)
##  latticeExtra   0.6-26  2013-08-15 CRAN (R 3.1.3)
##  magrittr       1.5     2014-11-22 CRAN (R 3.1.3)
##  maptools       0.8-37  2015-09-29 CRAN (R 3.1.3)
##  MASS           7.3-39  2015-02-24 CRAN (R 3.1.3)
##  Matrix         1.2-3   2015-11-28 CRAN (R 3.1.3)
##  MatrixModels   0.4-1   2015-08-22 CRAN (R 3.1.3)
##  mda            0.4-7   2015-05-25 CRAN (R 3.1.3)
##  memoise        1.0.0   2016-01-29 CRAN (R 3.1.3)
##  mgcv           1.8-4   2014-11-27 CRAN (R 3.1.3)
##  multcomp       1.4-1   2015-07-23 CRAN (R 3.1.3)
##  munsell        0.4.2   2013-07-11 CRAN (R 3.1.3)
##  mvtnorm        1.0-3   2015-07-22 CRAN (R 3.1.3)
##  nlme           3.1-120 2015-02-20 CRAN (R 3.1.3)
##  nnet           7.3-9   2015-02-11 CRAN (R 3.1.3)
##  plyr           1.8.3   2015-06-12 CRAN (R 3.1.3)
##  polspline      1.1.12  2015-07-14 CRAN (R 3.1.3)
##  polyclip       1.3-2   2015-05-27 CRAN (R 3.1.3)
##  pROC           1.8     2015-05-05 CRAN (R 3.1.3)
##  proto          0.3-10  2012-12-22 CRAN (R 3.1.3)
##  quantreg       5.19    2015-08-31 CRAN (R 3.1.3)
##  randomForest   4.6-12  2015-10-07 CRAN (R 3.1.3)
##  raster         2.4-20  2015-09-08 CRAN (R 3.1.3)
##  rasterVis      0.37    2015-09-06 CRAN (R 3.1.3)
##  RColorBrewer   1.1-2   2014-12-07 CRAN (R 3.1.3)
##  Rcpp           0.12.1  2015-09-10 CRAN (R 3.1.3)
##  reshape        0.8.5   2014-04-23 CRAN (R 3.1.3)
##  rmarkdown      0.9.2   2016-01-01 CRAN (R 3.1.3)
##  rms            4.4-0   2015-09-28 CRAN (R 3.1.3)
##  rpart          4.1-9   2015-02-24 CRAN (R 3.1.3)
```

```
##  sandwich       2.3-4   2015-09-24 CRAN (R 3.1.3)
##  scales         0.3.0   2015-08-25 CRAN (R 3.1.3)
##  sp           * 1.2-1   2015-10-18 CRAN (R 3.1.3)
##  SparseM        1.7     2015-08-15 CRAN (R 3.1.3)
##  spatstat       1.41-1  2015-02-27 CRAN (R 3.1.3)
##  stringi        1.0-1   2015-10-22 CRAN (R 3.1.3)
##  stringr        1.0.0   2015-04-30 CRAN (R 3.1.3)
##  survival     * 2.38-1  2015-02-24 CRAN (R 3.1.3)
##  tensor         1.5     2012-05-05 CRAN (R 3.1.3)
##  TH.data        1.0-6   2015-01-05 CRAN (R 3.1.3)
##  yaml           2.1.13  2014-06-12 CRAN (R 3.1.3)
##  zoo          * 1.7-12  2015-03-16 CRAN (R 3.1.3)
```
