# Supplementary material for: Modelling seasonal habitat suitability for wide-ranging species: Invasive wild pigs in northern Australia
Source: PLoS One. 2017 May 4;12(5):e0177018. doi: 10.1371/journal.pone.0177018 (PMC5417638; doi:10.1371/journal.pone.0177018)
Supplement: S1 Fig — (DOCX) [file pone.0177018.s005.docx]

**

**

**

**

**S1 Fig. Seasonal habitat suitability for wild pig breeding in the four validation backgrounds.** Presence records used for validation are shown for: Balkanu wet (A) and dry season (B), Lakefield wet (C) and dry (D) season, Oyala Thumotang wet season (E), NAQS dry season (F) and ALA wet (G) and dry (H) season. Descriptions of data sets are in Table 1. Validation backgrounds were defined from existing management units or by buffering data points.
