# Supplementary material for: Modelling seasonal habitat suitability for wide-ranging species: Invasive wild pigs in northern Australia
Source: PLoS One. 2017 May 4;12(5):e0177018. doi: 10.1371/journal.pone.0177018 (PMC5417638; doi:10.1371/journal.pone.0177018)
Supplement: S2 Fig — (DOCX) [file pone.0177018.s006.docx]

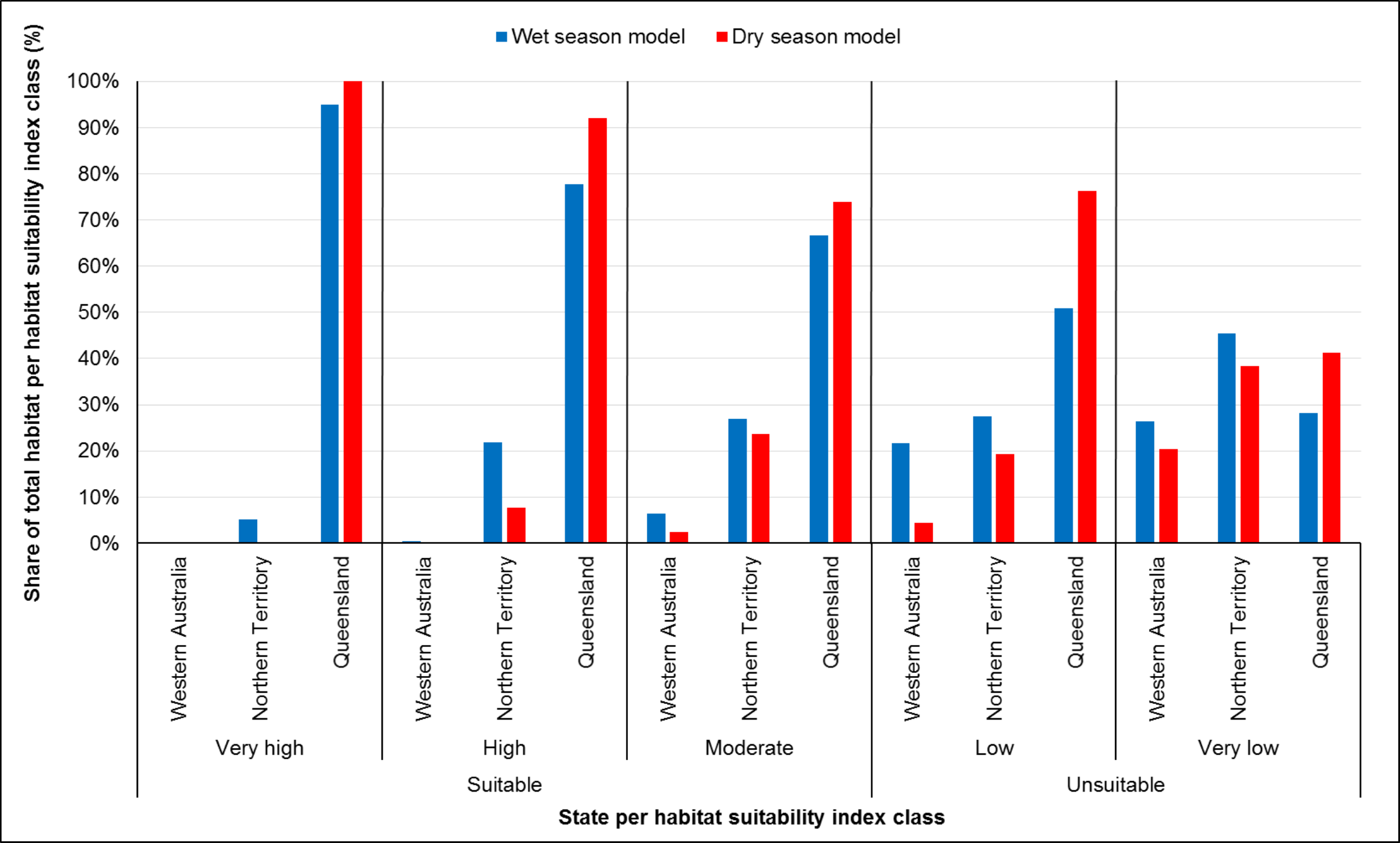


**S2.1 Fig. Share of seasonal habitat per state.** Percentages were calculated for each habitat suitability class separately. Location of states is shown in Fig 1.


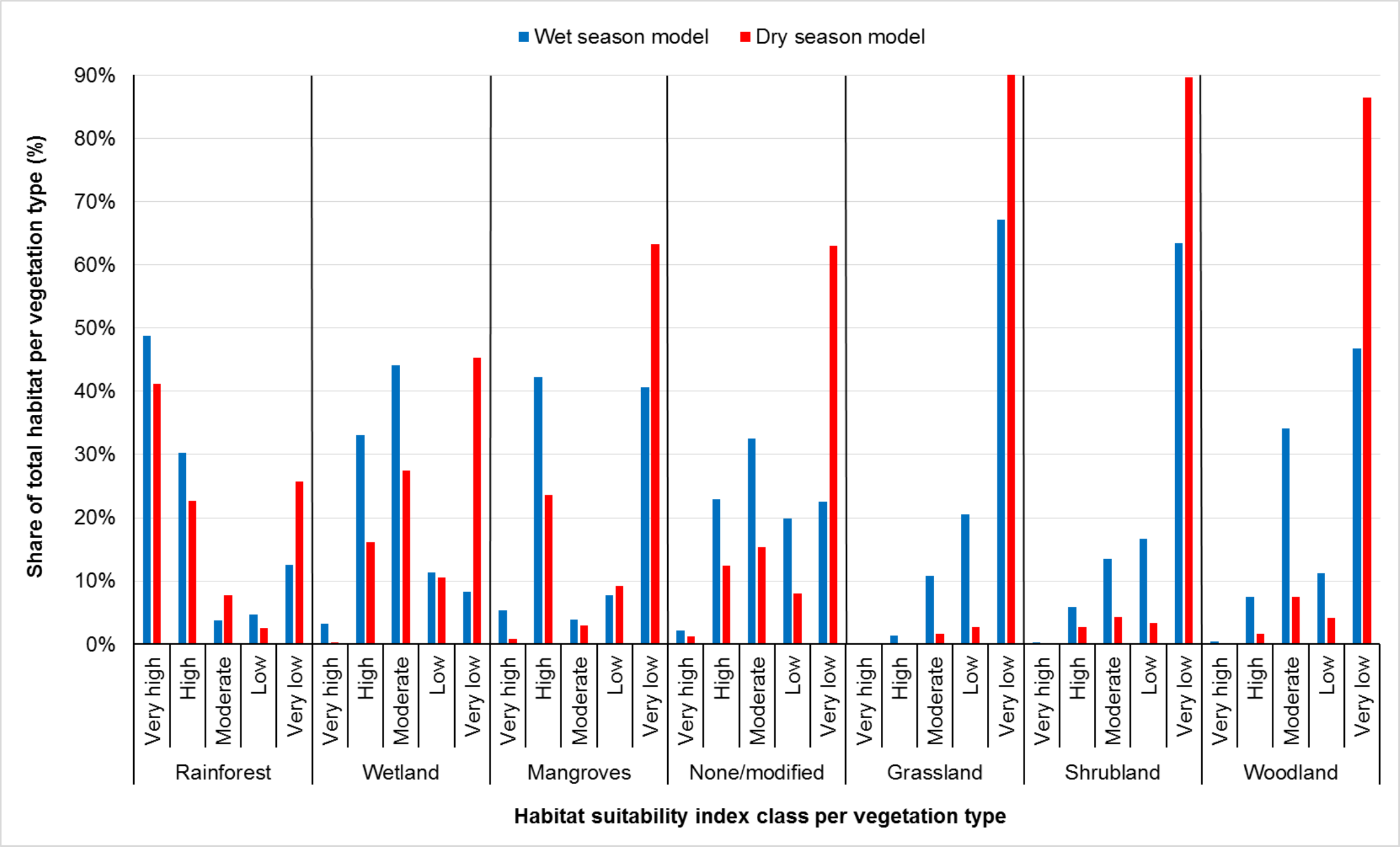


**S2.2 Fig. Distribution of modelled habitat suitability for each broad vegetation type.** Habitat suitability classes were taken from the Bayesian network model (S2.1 Table). Percentages were calculated separately for each vegetation type (from *Present Major Vegetation Groups* (MVG V.4.1).
